# Mingjing granule inhibits the subretinal fibrovascular membrane of two-stage laser-induced neovascular age-related macular degeneration in rats

**DOI:** 10.3389/fphar.2024.1384418

**Published:** 2024-06-25

**Authors:** Xiaoyu Li, Jiaxian Li, Weixin Zeng, Baoli Wang, Maobo Du, Lina Liang, Yun Gao

**Affiliations:** ^1^ Eye Hospital, China Academy of Chinese Medical Sciences, Beijing, China; ^2^ Ophthalmic Disease Project Group, China Evidence-based Medicine Center of Traditional Chinese Medicine, Beijing, China; ^3^ Institute of Chinese Materia Medica, China Academy of Chinese Medical Sciences, Beijing, China

**Keywords:** neovascular age-related macular degeneration, nAMD, Mingjing granule, two-stage laser, fibrovascular membrane, CNV

## Abstract

**Objective:**

The study aims to investigate the protective effect of Mingjing granule (MG) in a fibrovascular membrane rat model of neovascular age-related macular degeneration (nAMD) and explore the underlying mechanism.

**Methods:**

The nAMD fibrovascular membrane model was established by two-stage laser photocoagulation. BN rats were randomly divided into four groups: the model group was gavaged with distilled water, the anti-VEGF group was given an intravitreous injection of ranibizumab, the MG + anti-VEGF group was gavaged with MG combined with an intravitreous injection of ranibizumab, and the normal group not modeled only fed conventionally. Lesions were evaluated by color fundus photograph, optical coherence tomography, fundus fluorescein angiography, and retinal pigment epithelial–choroid–sclera flat mount. The changes in the retinal structure were observed by histopathology. The expression of inflammatory cell markers F4/80, Iba-1, and glial fibrillary acidic protein (GFAP); the fibrosis-related factors collagen-1, fibronectin, α-smooth muscle actin (α-SMA), and transforming growth factor-beta (TGF-β); and the complement system-related factors C3a and C3aR in the retina were detected by immunofluorescence or qRT-PCR.

**Results:**

The current study revealed that MG + anti-VEGF administration more significantly reduced the thickness of fibrovascular lesions, suppressed vascular leakage (exudation area and mean density value), inhibited the area of fibrovascular lesions, and restrained the formation of the fibrovascular membrane than the anti-VEGF agent alone in the two-stage laser-induced rat model. The fluorescence intensities of F4/80, Iba-1, collagen-1, fibronectin, TGF-β, and C3aR showed more significant inhibition in MG + anti-VEGF-treated rats than the anti-VEGF agent alone. The mRNA expression levels of F4/80, Iba-1, GFAP, collagen-1, fibronectin, α-SMA, TGF-β, and C3a showed lower levels in rats treated with MG + anti-VEGF than the anti-VEGF agent alone.

**Conclusion:**

Combining MG with anti-VEGF treatment inhibits the growth of the fibrovascular membrane more effectively than using anti-VEGF treatment alone. The mechanism underlying this effect may involve limiting inflammatory cell aggregation, controlling complement system activation, and decreasing the expression of the fibrotic protein.

## 1 Introduction

Age-related macular degeneration (AMD) is the leading cause of vision loss in the elderly over 50 (Liu and Liang, 2010), affecting nearly 200 million people worldwide (Spaide et al., 2020), which is mainly divided into two types, the dry and wet AMD. The latter, also known as neovascular AMD (nAMD), is a common form of advanced AMD, and its incidence is 15%–20% of that of AMD. Patients with nAMD will experience substantial visual loss in 80% of cases (Flaxel et al., 2020).

Choroidal neovascularization (CNV) is the main cause of visual loss in nAMD. CNV refers to etiologically diverse choroidal neovascularization sprouts that penetrate Bruch’s membrane and spread beneath the retinal pigment epithelial (RPE) or retinal neuroepithelial layer. Due to the structural and functional instability of neovascularization, blood and fluid leakage from tissue rupture leads to an inflammatory process with fibrotic changes, along with the release of pro- and anti-inflammatory cytokines, chemokines, and activation of the complement cascade reaction. Ultimately, retinal scarring damage or retinal detachment occurs, leading to decreased or loss of central vision (Witmer et al., 2003; Farnoodian et al., 2017; Martinez and Peplow, 2021).

The treatment for nAMD has undergone significant improvements over the past 2 decades. Almost all ophthalmologists consider anti-vascular endothelial growth factor (VEGF) medications to be the first-line treatment for nAMD as they can effectively promote the regression of CNV (Curtis et al., 2012). Ranibizumab (Lucentis) is the first anti-VEGF agent approved by the National Medical Products Administration of China and has been used in clinical treatment of nAMD since 2011. A multicenter, double-blind, randomized 2-year clinical trial (Anchor study) using a monthly regimen of ranibizumab (0.3 mg and 0.5 mg groups) compared to a traditional photodynamic therapy group to treat nAMD showed that the average visual acuity improvement was 8.5 letters in the ranibizumab 0.3 mg group, 11.3 letters in the ranibizumab 0.5 mg group, and 9.5 letters in the photodynamic therapy group. For the dominant classical CNV, ranibizumab treatment was better than photodynamic therapy (Brown et al., 2006). Despite the fact that anti-VEGF agents can typically stabilize or enhance vision, fibrotic scar formation has been identified as one of the causes of vision loss after treatment (Cohen et al., 2012).

Unfortunately, for some insensitive patients, anti-VEGF agents are not enough to control the recurrence of CNV and the further deterioration of vision caused by fibrotic scarring. Heier (2009) administered intravitreal ranibizumab once a month to patients with nAMD for 24 months. Then, after 2–6 years of follow-up, more than half of the patients developed macular fibrosis. Daniel et al. (2014) followed up patients with nAMD who received intravitreal injection of anti-VEGF agents according to the “3+prn” principle. After 2 years, nearly half of the affected eyes developed macular scars, resulting in poor vision. The above results found that the effect of anti-VEGF agents on macular fibrosis is limited, and the risk of complications associated with repeated intravitreal injections and the high cost of medical care impose a heavy psychological and financial burden on patients (Schmidt-Erfurth et al., 2014). Therefore, there is an urgent need for complementary and alternative therapies to preserve the characteristics of anti-VEGF agents while also supplementing their scope of action.

Mingjing granule (MG) is a formula for cooling blood and removing blood stasis in traditional Chinese medicine theory. It is widely used for nAMD patients with yin deficiency and blood stasis syndrome. It consists of eight kinds of botanical drugs, as shown in Table 1. Previous clinical studies have confirmed that MG improves the visual acuity of nAMD patients, diminishes the area of fundus bleeding and exudation, and inhibits the formation of CNV (Zhou et al., 2014a; Li et al., 2021). MG combined with anti-VEGF injection improves visual acuity and reduces the central retinal thickness and the number of injection times (Yu and Qin, 2020). In addition, the experimental results showed that MG could protect blue light-induced apoptosis in the human retinal pigment epithelium cultured *in vitro*, downregulate VEGF levels, and upregulate pigment epithelium-derived factor levels (Yu et al., 2008; Tang et al., 2009; Zhan, 2013). According to modern pharmacological research, the active metabolites of MG have hemostatic, anti-inflammatory, and immune-boosting properties (Feng et al., 2019). Therefore, we propose a hypothesis that MG and anti-VEGF agents may help treat the nAMD fibrovascular membrane.

**TABLE 1:** Formulation of MG.

Botanical name	Traditional name in Chinese	Produced from	Proportion (%) in daily prescription	Production batch number
*Typha angustifolia* L. [Typhaceae]	Puhuang	Dried pollen	13.04	20043161
*Eclipta prostrata* (L.) L. [Asteraceae]	Mohanlian	Dried aerial parts	17.39	20016041
*Astragalus mongholicus* Bunge [Fabaceae]	Huangqi	Dried root	26.09	20026161
*Salvia miltiorrhiza* Bunge [Lamiaceae]	Danshen	Dried root and rhizome	8.70	20031621
*Cirsium japonicum* DC. [Asteraceae]	Daji	Processed dried aerial parts	8.70	20025161
*Cirsium arvense* var. Arvense [Asteraceae]	Xiaoji	Dried aerial parts	13.04	20016911
*Lycium barbarum* L. [Solanaceae]	Gouqizi	Dried ripe fruit	4.34	22016111
*Ligustrum lucidum* W.T.Aiton [Oleaceae]	Nvzhenzi	Dried ripe fruit	8.70	20016941

We established a nAMD subretinal fibrovascular membrane model through a two-stage laser, referring to the method proposed by Karis, et al. (Little et al., 2020), in which a second laser burn was performed to induce CNV in order to induce leakage or bleeding. Compared to the conventional primary laser, the damage caused by the second laser is deeper, with a larger area and a higher fibronectin expression level. After 40 days following the second injury, the CNV transformed into a fibrovascular membrane. Establishing a fibrovascular membrane model is more appropriate for examining the pathogenesis of macular fibrosis and investigating therapeutic drugs.

In the current study, we used the nAMD fibrovascular membrane rat model to comprehensively evaluate the effects of MG combined with the anti-VEGF agent ranibizumab from histomorphological and molecular biological approaches, respectively, and tried to reveal the mechanism of action.

## 2 Materials and methods

### 2.1 Mingjing granule preparation

MG was produced by Beijing Tcmages Pharmaceutical Co., Ltd., China, and production batch numbers are listed in Table 1. The preparation methods of granules have been in accordance with the “Beijing Traditional Chinese Medicine Formula Granule Standards” issued by the Beijing Municipal Medical Products Administration. Botanical drugs (crude drugs) were taken, boiled with water, and filtered, and the filtrate was concentrated into a clear paste and dried (or crushed); then, an appropriate amount of excipients was added, mixed well, and made into granules. Then, granules of each single crude drug were mixed in a certain proportion to synthesize MG; then, 1 g of granule was approximately equivalent to 2.9 g of the crude drug. The formulation of MG and the content of each drug are shown in Table 1. The botanical drugs in MG are all covered in the Pharmacopoeia of the People’s Republic of China (Chinese Pharmacopoeia Commission, 2015). In addition, this study fully complies with the Nagoya Protocol and the Convention on International Trade in Endangered Species of Wild Fauna and Flora.

### 2.2 Reagents

The hematoxylin–eosin (H&E) kit was purchased from Beijing Yili Fine Chemicals Co., Ltd., China; ketamine hydrochloride injection (No: H20223609) was purchased from Zhejiang Jiuxu Pharmaceutical Co., Ltd., China; serazine hydrochloride injection (No: 20211001) was purchased from Dunhua Shengda Animal Medicine Co., Ltd., China; rat anti-F4/80 antibody (No: ab16911), rabbit anti-ionized calcium binding adapter molecule 1 (Iba-1) antibody (No: ab178846), collagen-1 rabbit antibody (No: ab270993), rabbit anti-fibronectin antibody (No: ab268020), rabbit anti-α-smooth muscle actin (α-SMA) antibody (No: ab280888), fluorescent secondary antibody goat anti-rat 488 (No: ab150157), and fluorescent second antibody goat anti-rabbit 594 (No: ab150077) were purchased from Abcam. Rabbit anti-transforming growth factor-beta 1 (TGF-β1) antibody (No: 21898) was purchased from Wuhan Sanying Biotechnology Co., Ltd., China; rabbit anti-C3aR antibody (No: bs2955R) was purchased from Beijing Boosen Biological Co., Ltd., China; DAPI antibody (No: AR1176) was purchased from Wuhan Borges De Biological Engineering Co., Ltd., China; RNeasy plus Universal Mini kits (No: 73404) were purchased from QIAGEN, Germany. iScript cDNA Synthesis Kit (No: 1708891) and iTaq Universal SYBR Green Supermix (No: 1725121) were purchased from Bio-Rad, United States.

The Novus Spectra DP frequency-doubled Nd: YAG laser therapy apparatus was manufactured by Lumenis, United States; the OPTO-RIS retinal imaging system was developed by Optoprobe Science Ltd., United Kingdom; Optical coherence tomography (Envisu R2210) and the automatic tissue dehydrator (ASP300S) were manufactured by Leica, Germany; the laser confocal scanning microscope (LSM880) was manufactured by ZEISS, Germany; and the PCR system (ABI7500) was developed by Applied Biosystems, United States.

### 2.3 Animals

Male BN rats (SPF, 8 weeks, 160–200 g) were purchased from Beijing Weitong Lihua Co., Ltd. (Beijing, China) with the experimental animal production license no: SCXK 2021-0006 and use license no: SYXK 2019-0049. All animals were housed in the laboratory animal center of Eye Hospital, Chinese Academy of Chinese Medical Sciences (CACMS) with a 12-h light/dark schedule, ambient air temperature of 22°C–23°C, and 45%–55% humidity. All rats had free access to standard laboratory rodent food and water. After 7-day conventional feeding for adaptation, the rats underwent anterior segment and fundus examination and those without ocular lesions were included in the experiment. This experiment complies with ARRIVE guidelines. All procedures were approved by the Institutional Animal Care and Use Committee of Eye Hospital, CACMS (Ethics Approval No. YKEC-DW-2022-001), performed in accordance with the Regulations for the Administration of Affairs Concerning Experimental Animals and the ARVO Statement for the Use of Animals in Ophthalmic and Vision Research.

### 2.4 Two-stage laser model of subretinal fibrosis

The rats were anesthetized with ketamine hydrochloride and serazine hydrochloride-mixed anesthetic (4 mL/kg) via intraperitoneal injection, pupils were dilated using compound tropicamide eye drops, and 0.4% aubucaine hydrochloride eye drops were used for local anesthesia of the ocular surface. A two-stage laser model was carried out as previously described (Little et al., 2020).

The first-stage laser: Krypton laser-induced laser burns were carried out in each eye at 3, 6, and 9 o’clock positions, between the blood vessels at the posterior pole of the fundus, approximately two disc diameters from the optic nerve. The parameters were set as spot diameter 100 µm, exposure time 0.1 s, and power 280 mW. After the laser was fully focused, one shot broke the Bruch’s membrane, accompanied by the generation of bubbles, which was the effective point. If the blood vessel was injured and the fundus bleeding was caused, or no bubbles were produced during the laser, it was the ineffective point. Ineffective points were considered modeling failures, they were excluded from the study, and animals were re-selected to establish a new model. The ineffective point of this study is less than 10%. The day of laser (CNV induction) was considered day −7 in the two-stage laser group of this study.

The second-stage laser: After 7 days (as day 0), the second laser burn was performed. The CNV lesions established 7 days ago were targeted by a laser beam (whitish/yellowish spots on the fundus without bleeding). Laser parameters were the same as the first laser, and secondary burns were performed. CNV and fibrovascular lesion were observed over the course of 40 days. Following animal welfare and ethical guidelines, only the right eye was selected to establish the model in this study.

### 2.5 Administration and grouping

MG was dissolved in distilled water by boiling (concentration: 1 g/mL); then, the solution was placed in a refrigerator at 4°C for storage. MG solution was intragastrically administered at a dose of 3.4 g/kg, and an equal volume of distilled water was used as the control. The results of previous studies have confirmed that this dose has the best effect on inhibiting choroidal neovascularization (Zhou et al., 2014b), so it was selected as the observation dose in this study. Gastric gavage started 1 day after the second stage of laser injury and lasted for 40 days. A single intravitreal injection of the anti-VEGF agent (ranibizumab) was performed 1 day after the second stage of laser injury. Compound anesthetic (chloral hydrate, serazine hydrochloride, and lidocaine) 3 mL/kg was injected intraperitoneally, pupils were dilated using compound tropicamide eye drops, and microforceps were used to help expose the eyeballs. The needle of the microsyringe was inserted into the rat eye from approximately 2 mm outside the corneal limbus of the temporal side along the direction of 45°. After the needle tip was seen in the pupil area, the rat eye was slowly injected with ranibizumab 5 μL/eye. After injection, we waited for 10 s and slowly pulled out the needle to prevent the outflow of liquid. After surgery, levofloxacin ointment was applied to the eyes.

Randomization was done by use of a computer-generated table of random numbers. The BN rats were randomly divided into 4 groups, 18 rats per group, an anti-VEGF group (rats with laser photocoagulation and intravitreous injection of ranibizumab), a MG + anti-VEGF group (rats with laser photocoagulation, intragastric administration of the MG solution, and intravitreous injection of ranibizumab), and a model group (rats with laser photocoagulation and intragastric administration of distilled water). Normal BN rats were not modeled and fed conventionally as a normal group. After intervention, researchers from non-animal breeders selected each group of animals using a table of random numbers method to complete the corresponding testing.

### 2.6 *In vivo* imaging

Lesions were observed at 20 and 40 days after the second stage of laser injury using fundus color photograph (FP), optical coherence tomography (OCT), and fundus fluorescein angiography (FFA). OCT scans were conducted using the Envisu R2210 OCT system. The fibrovascular membrane lesion was identified as a spindle-shaped hyperreflective region whose long axis paralleled with the level of the RPE. Multiple scans were taken across the entire lesion, and only the scan that passed through the center of the lesion was used to measure the thickness of fibrovascular membrane lesions, which was analyzed and calculated using Bioptigen Diver Image processing software and ImageJ software.

Fundus images and FFA were collected using the OPTO-RIS retinal imaging system. FFA images were captured within 5 min after intraperitoneal injection of 0.15–0.2 mL 10% sodium fluorescein. The exudation area (at 20 days, it was the vascular leakage; at 40 days, it was the staining of fibrovascular lesion) and mean density (MD) value of each lesion were measured using ImageJ software.

### 2.7 Retinal pigment epithelium–choroid–sclera flat mount staining

The eyes were enucleated from randomly selected rats at 40 days after the second stage of laser injury and fixed in 4% paraformaldehyde solution at 4°C for 24 h. The anterior segment and part of the vitreous were carefully removed under a microscope. The remaining eye cups were fixed in 4% paraformaldehyde solution for 1 h, and then, four scleral radial incisions were made. The retinal neuroepithelium layers were carefully removed with microforceps, and the remaining RPE–choroid–sclera complexes were made into flat mounts.

The RPE–choroid–sclera tissues were placed in a 24-well plate containing 0.25% Triton solution for 1h and then incubated with 2% BSA solution for 0.5 h. The 2% BSA solution was removed, and IB4 lectin was added according to the instructions. The 24-well plate was covered and incubated for 24 h at 4°C away from light. After washing with PBST for 10 min × 3 times, the RPE–choroid–sclera tissues were spread on the slides; anti-fluorescence attenuating tablets were dropped; and the cover slides were sealed, preserved at 4°C, and observed under a laser confocal microscope. The area of the lesions was calculated using ImageJ software.

### 2.8 Histopathology

Randomly selected rats were sacrificed by excessive anesthesia at 40 days after the second stage of laser injury, and thereafter, the eyes were promptly enucleated and immersed in Davidson’s fixative for 3 h. The anterior segment and part of the vitreous were carefully removed under a microscope. The remaining eye cups were dehydrated in an automatic tissue dehydrator and then embedded in paraffin. Sections (4 μm) were made, and H&E staining was carried out using the standard protocol. The pathological changes of the retina were observed using an optical microscope.

### 2.9 Immunofluorescence staining

The eyes were enucleated from randomly selected rats at 40 days after the second stage of laser injury and fixed in 4% paraformaldehyde solution at 4°C. After OCT embedding, 8-μm frozen sections were made and stored in a refrigerator at −20°C. The frozen sections were eluted with PBS at room temperature, then placed in 1% TritonX-100 solution for 30 min, followed by PBS for 15 min × 2 times, 2% BSA was quickly added, and the reaction was performed in a 37°C water bath for 30 min to block non-specific antigens. Sections were incubated with primary antibodies (F4/80, Iba-1, GFAP, collagen-1, fibronectin, α-SMA, TGF-β, and C3a) overnight at 4°C, followed by PBS for 10 min × 3 times, and the second antibodies were added and incubated at 40°C for 45 min. The nuclei were counterstained with DAPI for 10 min at room temperature after washing with PBS, and sections were sealed with an anti-fluorescence attenuating tablet. A fluorescence microscope was used to observe the expression position and relative expression level of fibrovascular membrane-related proteins in the macular area. The mean fluorescence intensity was recorded using Image Pro Plus software.

### 2.10 Quantitative real-time PCR

Total RNA was extracted using the TRIzol extraction kit, and the RNA concentration was determined using an ultramicro spectrophotometer. RNA was synthesized into a cDNA template by reverse transcription according to the instructions of the iScript cDNA Synthesis Kit. The primers were synthesized by Beijing Dingguo Changsheng Biotechnology Co., Ltd., and the sequences of primers are shown in Table 2. PCR products were amplified using the 7500 Fast Real-Time PCR System. All samples should be tested three times, and the data should be saved for subsequent statistical calculation. GAPDH was used as the internal reference gene. PCR automatic analysis software was used for fluorescence data analysis. The comparison Ct method was used to detect F4/80, Iba-1, glial fibrillary acidic protein (GFAP), collagen-1, fibronectin, α-SMA, TGF-β, C3a, and C3aR in each group.

**TABLE 2:** Primer sequences of qRT-PCR.

Primer name	Sequence
Collagen-1-forward	GAT​GGA​CTC​AAC​GGT​CTC​CC
Collagen-1-reverse	CGG​CCA​CCA​TCT​TGA​GAC​TT
Fibronectin-forward	ATG​AGA​AGC​CTG​GAT​CCC​CT
Fibronectin-reverse	GGA​AGG​GTA​ACC​AGT​TGG​GG
TGF-β-forward	GAC​TCT​CCA​CCT​GCA​AGA​CC
TGF-β-reverse	GGA​CTG​GCG​AGC​CTT​AGT​TT
C3A-forward	TCT​GCC​TAT​GCT​GCC​TTC​AA
C3A-reverse	ATC​ACT​GGT​CCG​TCC​TCC​T
C3Ar1 F-forward	TCC​TCT​GCT​GCC​TCT​CCT​T
C3Ar1 F-reverse	GTT​CTC​ACG​CTC​CGT​AGG​T
F4/80-forward	CTC​TTC​TGG​GGC​TTC​AGT​GG
F4/80-reverse	CAG​GTG​GCA​TAA​GCT​GGA​CA
Iba-1-forward	CAG​CCT​CAT​CGT​CAT​CTC​CC
Iba-1-reverse	TTC​CTG​TTG​GGC​TTT​CAG​CA
GFAP-forward	TGC​ATG​TAC​GGA​GTA​TCG​CC
GFAP-reverse	GGG​GGA​GGA​AAG​GAC​AAC​TG
α-SMA-forward	ACC​ATC​GGG​AAT​GAA​CGC​TT
α-SMA-reverse	CTG​TCA​GCA​ATG​CCT​GGG​TA

### 2.11 The method of fingerprint analysis of MG

Chromatographical analysis was conducted on the Waters e2695 HPLC platform (Waters Company, United States; PDA Detector, Empower 3 workstation), and methanol was purchased from Beijing Chemical Works, China. Acetonitrile was purchased from Thermo Fisher Scientific Co., Ltd., United States. Phosphoric acid was purchased from Tianjin Guangfu Technology Development Co., Ltd., China.

Salidroside (PS011472), typhaneoside (PS000961), calycosin-7-glucoside (PS000687), rutin (PS012233), isorhamnetin-3-O-neohespeidoside (PS011873), salvianolic acid B (PS012387), wedelolactone (PS011796), linarin (PS011370), pectolinarin (PS000267), and astragaloside IV (PS012327) were purchased from Chengdu Push Bio-technology Co., Ltd., China.

Ten batches of MG were used in this study, namely, S1–S10, and control spectrum R. Specimens of MG were deposited in the Eye Hospital China Academy of Chinese Medical Sciences. MG (7.93 g) was weighed and then extracted under ultrasound with methanol (50 mL). The solvent was collected after filtration. Samples were subjected to separation using the Agilent HC-C_18_ chromatographic column (4.6 mm × 250 mm, 5 μm). The mobile phase was acetonitrile (A) and 0.1% phosphoric acid aqueous solution (B), and gradient elution was performed according to the elution procedure in Table 3. The flow rate was 1.0 mL/min, the column temperature was 30°C, and the detection wavelength was 254 nm.

**TABLE 3:** Gradient elution procedure.

Time (min)	Mobile phase A (%)	Mobile phase B (%)
0	10	90
15	15	85
30	25	75
50	30	70
55	70	30
60	100	0
65	100	0

### 2.12 Statistical analysis

All measurements were performed in a blinded fashion. Data were expressed as mean ± standard deviation (*‾x ± s*). Data were tested for normality, and variances were tested to ensure similarity. The data conforming to normal distribution and homogeneity of variance were compared using one-way ANOVA, and the LSD-t test was used for pairwise comparison. Independent samples’ *t*-test was used to compare the means of two groups with normal distribution. Differences were considered statistically significant when the *p*-value <0.05. GraphPad Prism v. 8.0.1 software was used to create graphs and conduct statistical analysis.

## 3 Results

### 3.1 Establishment of the nAMD fibrovascular membrane model

**FIGURE 1:**
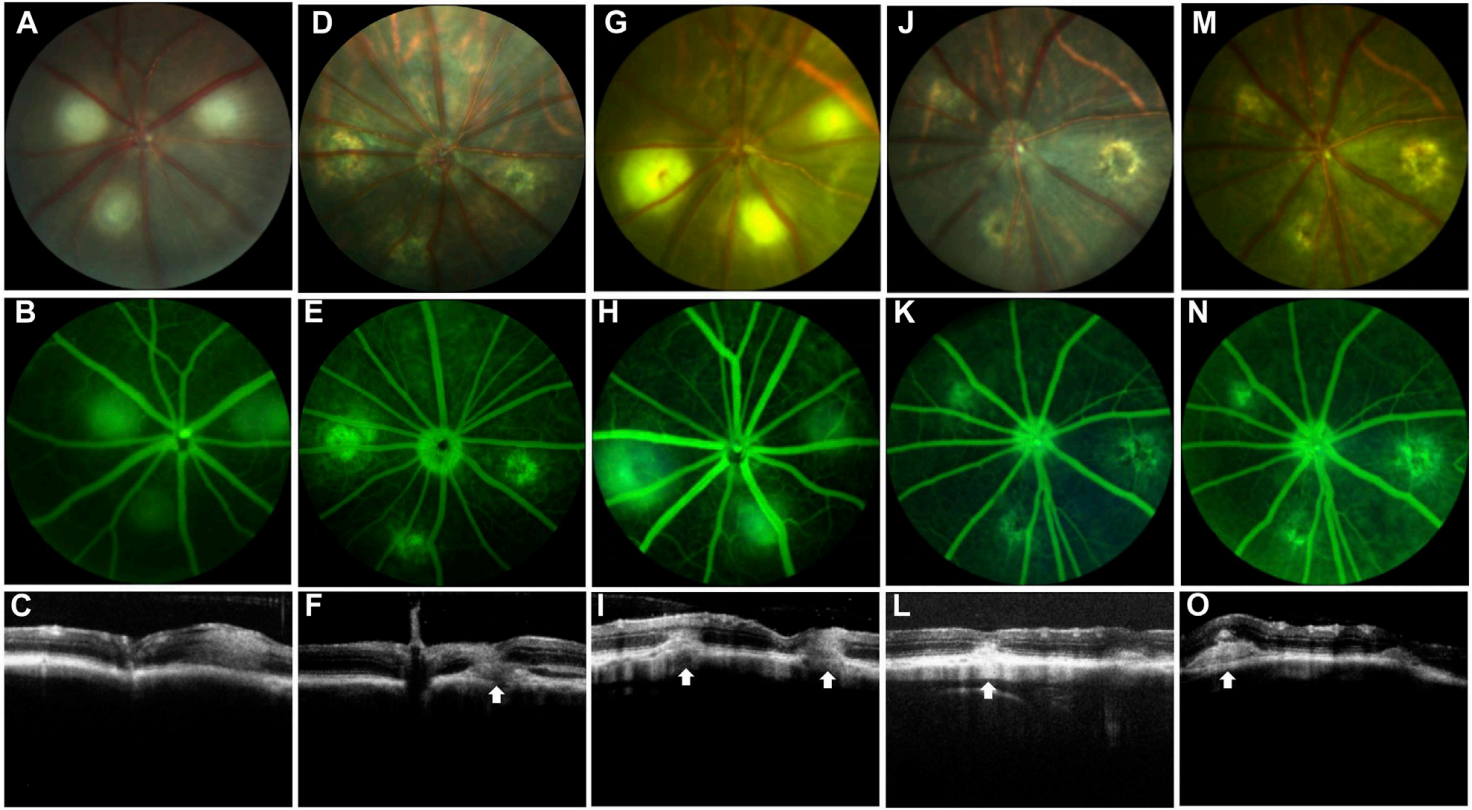
Establishment of the nAMD fibrovascular membrane model. FP, FFA, and OCT images at different time points. White arrows depict the center of the lesion. The first row shows the image of FP, the second row shows the image of FFA, and the third row shows the image of OCT. **(A–C)** Images shown were captured at day 1 and **(D–F)** were captured day 7 after the first laser; **(G–I)** images shown were captured at day 1, **(J–L)** were captured at day 20, and **(M–O)** images shown were captured at day 40 after the second laser; *n* = 15.

In the current study, a rat model of subretinal fibrosis was established by a two-stage laser. FP, FFA, and OCT were used to observe the changes of retinal lesions in living animals. FP showed that krypton laser photocoagulation caused the rupture of Bruch’s membrane, and the laser lesions showed grayish edema (Figures 1A–C). No bleeding was observed in the laser lesions following the first laser photocoagulation; however, bleeding was noticed in certain places after 7 days (Figure 1D). FFA and OCT results revealed that 1 day after the first laser, only minor local prominence of the lesion was observed, and no significant leakage occurred (Figures 1B, C). After 7 days, some small area of fluorescence leakage appeared in the laser lesion area, and CNV and subretinal effusion were observed (Figures 1E, F).

The extent and thickness of the laser lesions had expanded 1 day after the second laser, and CNV and fluorescence leakage were seen (Figures 1G–I). Twenty days later, CNV clusters with fluorescence leaking were observed, and the thickness of the lesions increased (Figures 1J–L). The extent of the lesion and thickness further developed after 40 days following the second laser compared to 20 days, fluorescein staining of fibrovascular lesions was shown at the lesion. The subretinal fibrous vascular membrane was formed (Figures 1M–O).

### 3.2 MG reduced vascular leakage and formation of the fibrovascular membrane

**FIGURE 2:**
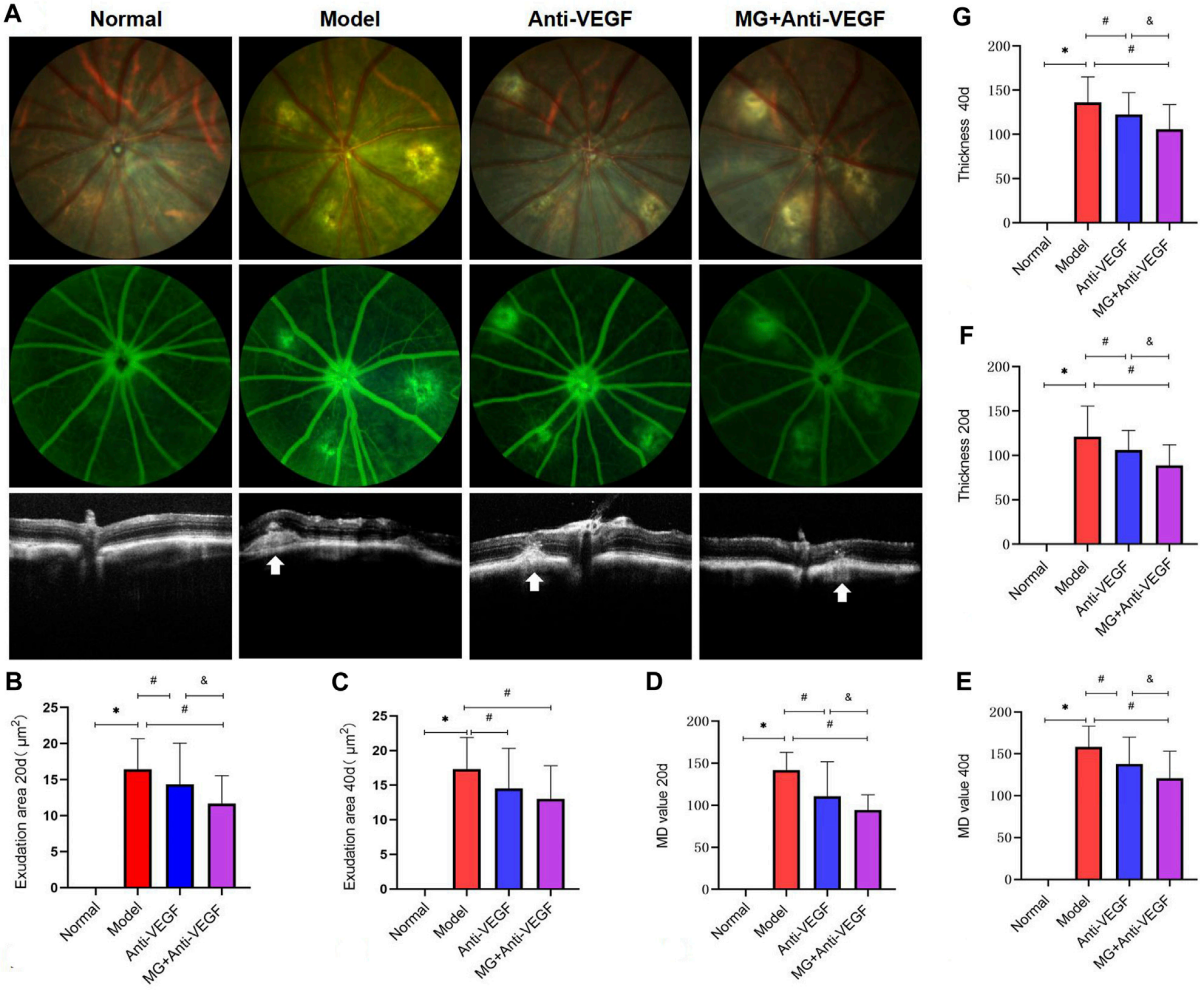
Morphological effect of MG on vascular leakage in the fibrovascular membrane model. White arrows depict the center of the lesion. * model vs. normal; # anti-VEGF/MG + anti-VEGF vs. model; and &MG + anti-VEGF vs. anti-VEGF; data are represented as mean ± SD; FP, FFA, and OCT: *n* = 15. **(A)** FP, FFA, and OCT imaging at 40 days after the second laser. The first row shows the image of FP, the second row shows the image of FFA, and the third row shows the image of OCT. Each column represents a group. **(B)** Exudation area at 20 days: *p = 0.000 and #*p* = 0.038 (anti-VEGF) and *p* = 0.000 (MG + anti-VEGF), &*p* = 0.020, and **(C)** at 40 days after the second laser: *p = 0.000, #*p* = 0.013 (anti-VEGF), and *p* = 0.000 (MG + anti-VEGF). **(D)** MD value at 20 days: *p = 0.000 and #*p* = 0.000 (anti-VEGF), and *p* = 0.000 (MG + anti-VEGF), &*p* = 0.045, and **(E)** at 40 days after the second laser: *p = 0.000 and #*p* = 0.003 (anti-VEGF) and *p* = 0.000 (MG + anti-VEGF), &*p* = 0.023. **(F)** Thickness of lesions at 20 days: *p = 0.000, #*p* = 0.027 (anti-VEGF), and *p* = 0.000 (MG + anti-VEGF), &*p* = 0.014, and **(G)** at 40 days after the second laser: *p = 0.000, #*p* = 0.038 (anti-VEGF) and *p* = 0.000 (MG + anti-VEGF), &*p* = 0.017.

In order to determine the role of MG in the nAMD fibrovascular membrane, FFA was used to compare the pathological vascular leakage between the different groups at 20 days after the second laser and compare the staining of fibrovascular lesion at 40 days after the second laser. The results demonstrated that the group with administration of MG + anti-VEGF had fewer and smaller areas of vascular leakage and fluorescent staining than the group without treatment or with single anti-VEGF (Figure 2A).

Quantitative analysis showed that the exudation area (Figures 2B, C) of the anti-VEGF group (at 20 days: *p* = 0.038; at 40 days: *p* = 0.013) and the MG + anti-VEGF group (at 20 days: *p* = 0.000; at 40 days: *p* = 0.000) significantly decreased as compared to the model group at 20 and 40 days after the second laser. However, the MG + anti-VEGF group was significantly decreased compared with the anti-VEGF group (*p* = 0.020) at 20 days after the second laser, but there was no statistical difference at 40 days (*p =* 0.227).

The MD value (Figures 2D, E) of the anti-VEGF group (at 20 days: *p* = 0.000; at 40 days: *p* = 0.003) and the MG + anti-VEGF group (at 20 days: *p* = 0.000; at 40 days: *p* = 0.000) significantly decreased as compared to the model group at 20 and 40 days after the second laser. The MG + anti-VEGF group was significantly decreased compared with that of the anti-VEGF group (at 20 days: *p* = 0.045; at 40 days: *p* = 0.023).

### 3.3 MG attenuated the formation of the fibrovascular membrane, as analyzed by OCT

OCT was performed to measure the thickness of lesions (Figure 2). Whether it was 20 (Figure 2F) or 40 (Figure 2G) days after the second laser, we found that the thickness of lesions was significantly reduced after the treatment of anti-VEGF (at 20 days: *p* = 0.027; at 40 days: *p* = 0.038) or MG + anti-VEGF (at 20 days: *p* = 0.000; at 40 days: *p* = 0.000) compared with the model group. The MG + anti-VEGF group was significantly decreased compared with the anti-VEGF group (at 20 days: *p* = 0.014; at 40 days: *p* = 0.017).

### 3.4 MG reduced the lesion area in the fibrovascular membrane model

**FIGURE 3:**
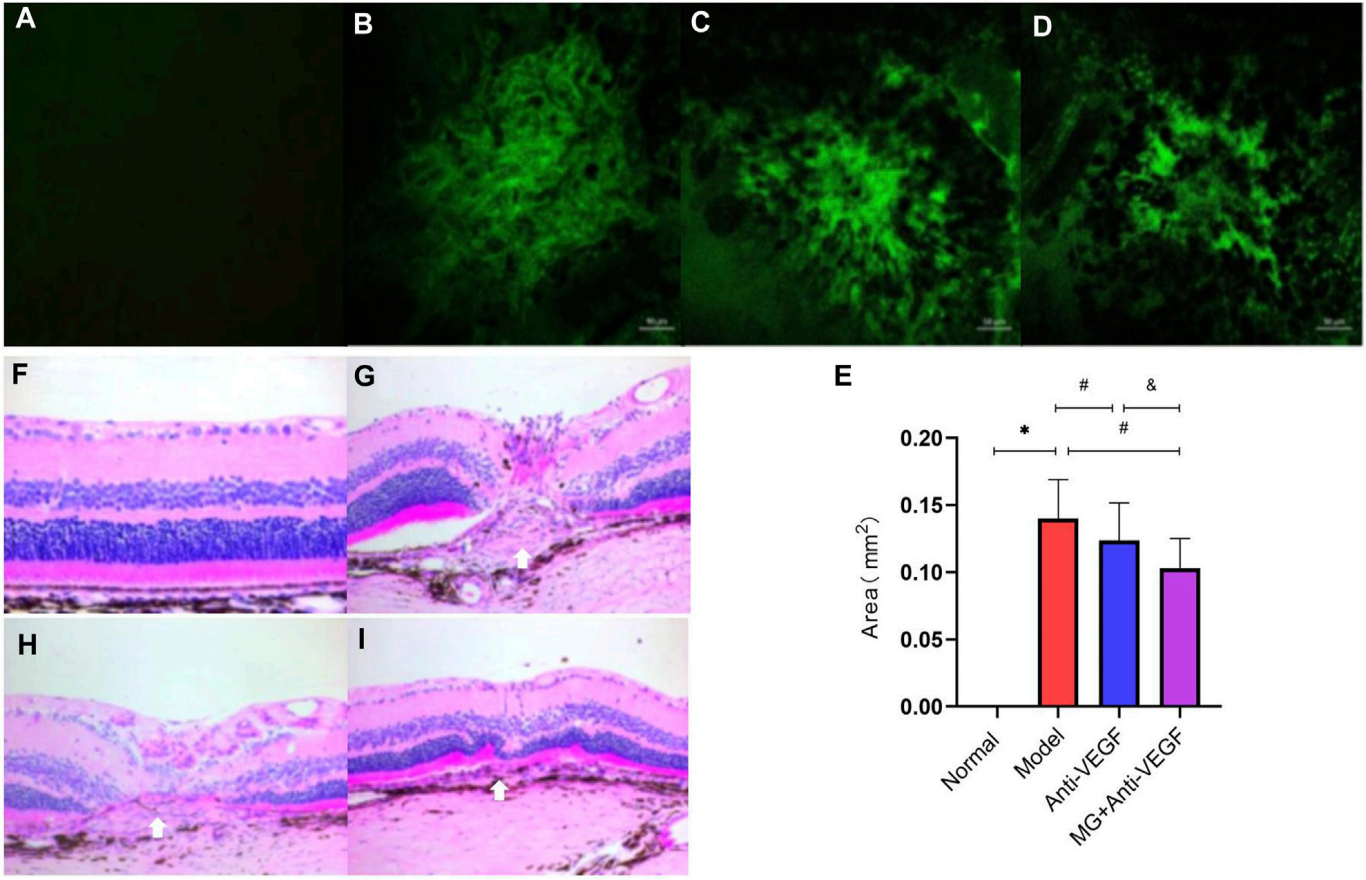
Histopathologic effect of MG on the fibrovascular membrane. White arrows depict the center of the lesion. **(A–D)** RPE–choroid–sclera flat mounts were made from the rats and stained with IB4 (green); **(A)** normal group; **(B)** model group; **(C)** anti-VEGF group; and **(D)** MG + anti-VEGF group. **(E)** Histogram of the lesion area; * model vs. normal, *p* = 0.000; # anti-VEGF vs. model, *p* = 0.012, and MG + anti-VEGF vs. model, *p* = 0.000; and &MG + anti-VEGF vs. anti-VEGF, *p* = 0.004; data are represented as mean ± SD; *n* = 5. **(F–I)**: H&E staining of the fibrovascular membrane lesion (x20). **(F)** Normal group; **(G)** model group; **(H)** anti-VEGF group; and **(I)** MG + anti-VEGF group.

To further study the effect of MG on fibrovascular membrane formation, RPE–choroid–sclera flat mounts were used to measure the lesion area. On day 40 after the second laser, RPE–choroid–sclera tissues were made into flat mounts, which were stained by immunofluorescence labeling with IB4, and the images of lesion were captured using a confocal laser microscope (Figures 3A–D). Then, the lesion area was quantified using ImageJ software (Figure 3E). Compared with the model group, anti-VEGF (*p* = 0.012) and MG + anti-VEGF groups (*p* = 0.000) were significantly decreased. However, the MG + anti-VEGF group had a lower value than the anti-VEGF group (*p* = 0.004).

### 3.5 MG alleviated lesion as analyzed by H&E staining in the fibrovascular membrane model

H&E staining showed that the retina in the normal group had a complete structure with a clean, orderly arrangement (Figure 3F). At 40 days after the second laser, the structure of the retina at the laser spot was abnormal, with a chaotic retinal structure of each layer, and the lesions were located between photoreceptors and choroid, that is, subretinal space. The center of the laser spot had a concave appearance, with edema and protrusions on both sides. The fibrovascular membrane lesion could be observed between the RPE layer and the outer nuclear layer, with CNV infiltrating the outer nuclear layer (Figure 3G). The severity of retinal structural damage in the anti-VEGF group was much lower than that in the model group (Figure 3H). The MG + anti-VEGF group has the least damage to the retinal structure (Figure 3I).

### 3.6 MG inhibited the activation of inflammatory cells

Macrophage-, microglia-, and astrocyte-mediated inflammatory responses play a non-negligible and important role in the pathogenesis of nAMD. In order to quantify the expression of related markers in the retina, we labeled the macrophage marker F4/80, the microglia marker Iba-1, and the astrocyte marker GFAP with immunofluorescence at 40 days after the second laser. qRT-PCR was also used to determine the relative expression levels of mRNA for these factors in retinal choroidal tissue. After the second laser, we detected a noticeable upregulation of the expression levels of F4/80, Iba-1, and GFAP.

**FIGURE 4:**
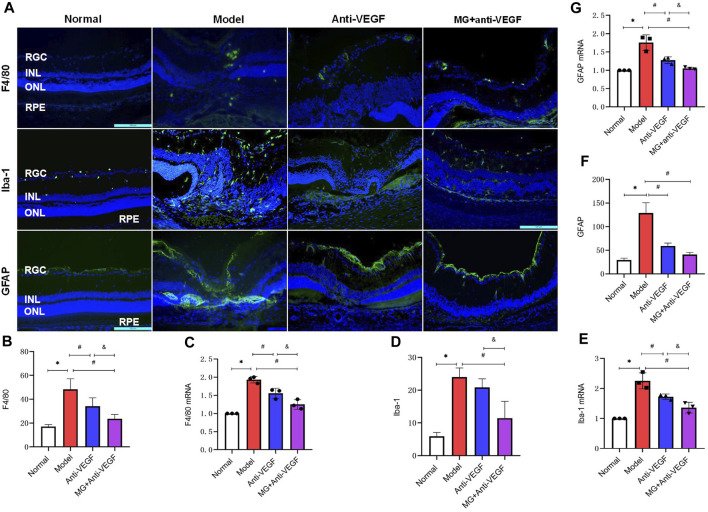
MG inhibited the activation of inflammatory cells. RGC, retinal ganglion cell layer; INL, inner nuclear layer; ONL, outer nuclear layer; RPE, retinal pigment epithelial layer; * model vs. normal; # anti-VEGF/MG + anti-VEGF vs. model; and &MG + anti-VEGF vs. anti-VEGF; data are mean ± SD; immunofluorescence: *n* = 5; qRT-PCR: *n* = 3. **(A)** Image of the immunofluorescence staining, rows represent different factors, and columns represent different groups. Immunofluorescence expression levels of **(B)** F4/80: *p = 0.000, #*p* = 0.003 (anti-VEGF) and *p* = 0.000 (MG + anti-VEGF), &*p* = 0.023; **(D)** Iba-1: *p = 0.000, #*p* = 0.000 (MG + anti-VEGF), &*p* = 0.005; **(F)** GFAP: *p = 0.000, #*p* = 0.000 (anti-VEGF) and *p* = 0.000 (MG + anti-VEGF). Expression levels of **(C)** F4/80 mRNA: *p = 0.000, #*p* = 0.003 (anti-VEGF) and *p* = 0.000 (MG + anti-VEGF), &*p* = 0.008, **(E)** Iba-1 mRNA: *p = 0.000, #*p* = 0.005 (anti-VEGF) and *p* = 0.000 (MG + anti-VEGF), &*p* = 0.028, **(G)** GFAP mRNA: *p = 0.000, #*p* = 0.001 (anti-VEGF) and *p* = 0.000 (MG + anti-VEGF), &*p* = 0.047.

F4/80+ cells were expressed only in small amounts in the normal retina, and after laser modeling, F4/80-labeled cells were seen to be distributed in large numbers between the retinal ganglion cell layer to the inner nuclear layer, and after treatment with either anti-VEGF or MG + anti-VEGF, the F4/80+ cells were reduced and distributed only in small numbers in the ganglion cell layer (Figure 4A). Quantitative analysis of fluorescence intensity showed that both the anti-VEGF (*p* = 0.003) and MG + anti-VEGF groups (*p* = 0.000) were significantly lower than the model group, and the MG + anti-VEGF group was significantly lower than the anti-VEGF group (*p* = 0.023) (Figure 4B). qRT-PCR results were in accordance with immunofluorescence findings, and the MG + anti-VEGF group’s relative expression level of F4/80 mRNA was lower than that of the anti-VEGF group (*p* = 0.008) (Figure 4C).

Iba-1+ cells were only expressed in the ganglion cell layer of the normal retina, but after laser modeling, it was shown that Iba-1+ cells were spread throughout the retina’s layers, with the fibrovascular membrane lesions having the densest distribution. Treatments with anti-VEGF or MG + anti-VEGF decreased the proportion of Iba-1+ cells, which were sparsely dispersed between the ganglion cell layer and the nerve fiber layer (Figure 4A). Quantitative analysis of fluorescence intensity showed that the MG + anti-VEGF group was significantly lower than the model group (*p* = 0.000), and there was no statistical difference between the anti-VEGF and model groups (*p* = 0.276) (Figure 4D). However, the qRT-PCR results indicated that both the anti-VEGF (*p* = 0.005) and MG + anti-VEGF groups (*p* = 0.000) were significantly lower than the model group, and the MG + anti-VEGF group was significantly lower than the anti-VEGF group (*p* = 0.028) (Figure 4E).

In the normal retina, GFAP + cells were distributed throughout the inner limiting membrane. After the second laser, GFAP+ was observed to be concentrated at the inner limiting membrane and at the inner nuclear layer lesions. Following treatment with either anti-VEGF or MG + anti-VEGF, GFAP + cells were only visible in the inner limiting membrane (Figure 4A). According to a quantitative analysis of the data, the anti-VEGF (*p* = 0.000) and MG + anti-VEGF groups (*p* = 0.000) both had significantly lower fluorescence intensities than the model group. When MG + anti-VEGF was compared to the anti-VEGF group, there was no statistically significant difference (*p* = 0.066) (Figure 4F). However, qRT-PCR results showed that the relative expression level of GFAP mRNA was lower in the MG + anti-VEGF group than that in the anti-VEGF group (*p* = 0.047) (Figure 4G).

### 3.7 MG delayed the process of fibrosis

Since collagen-1, fibronectin, α-SMA, and TGF-β are all extensively involved in nAMD fibrosis, immunofluorescence and qRT-PCR were used to map their distribution and measure the level of expression in the retina. After the second laser, there was a noticeably increased expression of collagen-1, fibronectin, α-SMA, and TGF-β.

**FIGURE 5:**
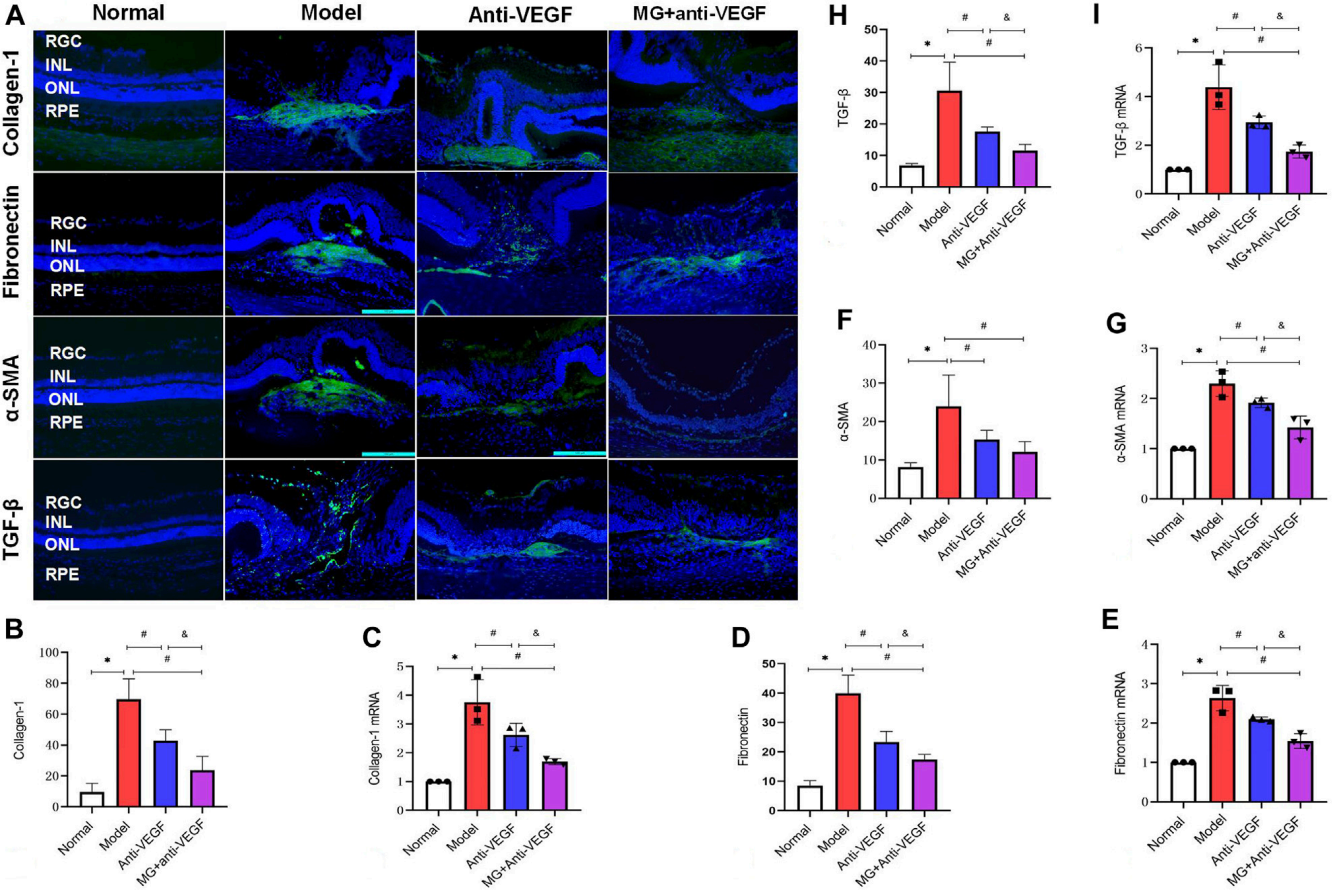
MG delayed the process of fibrosis. RGC, retinal ganglion cell layer; INL; inner nuclear layer; ONL, outer nuclear layer; RPE; retinal pigment epithelial layer; * model vs. normal; # anti-VEGF/MG + anti-VEGF vs. model; and &MG + anti-VEGF vs. anti-VEGF; data are represented as mean ± SD; immunofluorescence: *n* = 5; qRT-PCR: *n* = 3. **(A)** Image of immunofluorescence staining, rows represent different factors, and columns represent different groups. Immunofluorescence expression levels of **(B)** collagen-1: *p = 0.000, #*p* = 0.004 (anti-VEGF) and *p* = 0.000 (MG + anti-VEGF), &*p* = 0.014; **(D)** fibronectin: *p = 0.000, #*p* = 0.000 (anti-VEGF) and *p* = 0.000 (MG + anti-VEGF), &*p* = 0.021; **(F)** α-SMA: *p = 0.000, #*p* = 0.010 (anti-VEGF) and *p* = 0.001 (MG + anti-VEGF); **(H)** TGF-β: *p = 0.000, #*p* = 0.001 (anti-VEGF) and *p* = 0.000 (MG + anti-VEGF), &*p* = 0.038. Expression levels of **(C)** collagen-1 mRNA: *p = 0.000, #*p* = 0.014 (anti-VEGF) and *p* = 0.001 (MG + anti-VEGF), &*p* = 0.034; **(E)** fibronectin mRNA: *p = 0.000, #*p* = 0.008 (anti-VEGF) and *p* = 0.000 (MG + anti-VEGF), &*p* = 0.007; **(G)** α-SMA mRNA: *p = 0.000, #*p* = 0.030 (anti-VEGF) and *p* = 0.000 (MG + anti-VEGF), &*p* = 0.010; **(I)** TGF-β mRNA: *p = 0.000, #*p* = 0.007 (anti-VEGF) and *p* = 0.000 (MG + anti-VEGF), &*p* = 0.018.

In normal eyes, collagen-1+ cells were seen in the scleral tissues. Following the second laser, these cells were centrally expressed at fibrovascular membrane lesions (Figure 5A). Quantitative measurement of fluorescence intensity revealed that it was significantly lower in the anti-VEGF (*p* = 0.004) and the MG + anti-VEGF groups (*p* = 0.000) than the model group. In comparison to the anti-VEGF group, the MG + anti-VEGF group showed a significant reduction (*p* = 0.014) (Figure 5B). qRT-PCR validated the results of immunofluorescence. Similarly, the MG + anti-VEGF group had the lowest relative expression level of collagen-1 mRNA compared to the model (*p* = 0.001) and anti-VEGF group (*p* = 0.034) (Figure 5C).

Normal retinal fibronectin + cell distribution is uncommon, and following laser injury, strong expression was observed at the fibrovascular membrane lesions and migrated to the inner layers of the retina along with the injury sites (Figure 5A). Both the anti-VEGF (*p* = 0.000) and MG + anti-VEGF groups (*p* = 0.000) had significantly lower fluorescence intensities than the model group, according to quantitative analyses. Compared to the anti-VEGF group, the MG + anti-VEGF group showed a significant decline (*p* = 0.021) (Figure 5D). qRT-PCR confirmed the immunofluorescence findings, and the MG + anti-VEGF group had the lowest relative fibronectin mRNA expression level compared to the model (*p* = 0.000) and anti-VEGF group (*p* = 0.007) (Figure 5E).

The normal retina had few α-SMA + cells, whereas following laser injury, fibrovascular membrane lesions showed strong expression (Figure 5A). According to a quantitative analysis of the data, the anti-VEGF (*p* = 0.010) and MG + anti-VEGF groups (*p* = 0.001) both had significantly lower fluorescence intensities than the model group. When compared to the anti-VEGF group, there was no significant variance between the MG + anti-VEGF and anti-VEGF groups (*p* = 0.259) (Figure 5F). However, qRT-PCR findings indicated that when compared to the model (*p* = 0.000) and anti-VEGF groups (*p* = 0.010), the MG + anti-VEGF group’s relative expression level of α-SMA mRNA was the lowest (Figure 5G).

TGF-β+ cells were sparsely distributed in the normal retina and followed the fibrovascular membrane lesions through the whole retina after laser injury. TGF-β+ cells were diminished after anti-VEGF or MG + anti-VEGF therapy and were distributed only sparingly in the fibrovascular membrane lesions (Figure 5A). Quantitative analysis of fluorescence intensity showed that both the anti-VEGF (*p* = 0.001) and MG + anti-VEGF groups (*p* = 0.000) were significantly lower than the model group. Compared to the anti-VEGF group, the MG + anti-VEGF group showed a significant decline (*p* = 0.038) (Figure 5H). qRT-PCR validated the results, and TGF-β mRNA relative expression levels were lowest in the MG + anti-VEGF group compared to the model (*p* = 0.000) and anti-VEGF groups (*p* = 0.018) (Figure 5I).

### 3.8 The effect of MG on the complement system

The complement system is crucial in the development of CNV. C3 is a vital part of the complement system, and C3a, which is created when C3 is activated, forms the basis of the complement-dependent reaction. As a consequence, we used an immunofluorescence assay to mark the distribution of C3aR (C3a receptor) in the retina and applied qRT-PCR to semiquantitatively measure the expression levels of C3a and C3aR mRNA in the retina. C3a and C3aR expression levels were observed to be extremely upregulated following the second laser.

**FIGURE 6:**
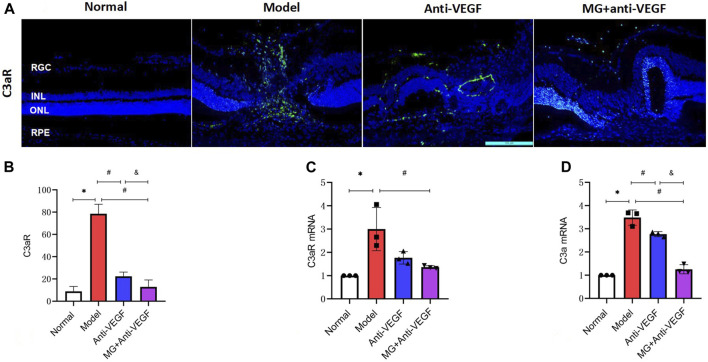
Effect of MG on the complement system. RGC, retinal ganglion cell layer; INL, inner nuclear layer; ONL, outer nuclear layer; RPE, retinal pigment epithelial layer; * model vs. normal; # anti-VEGF/MG + anti-VEGF vs. model; and &MG + anti-VEGF vs. anti-VEGF; data are represented as mean ± SD; immunofluorescence: *n* = 5; qRT-PCR: *n* = 3. **(A)** Image of immunofluorescence staining, rows represent different factors, and columns represent different groups. **(B)** Immunofluorescence expression levels of C3aR: *p = 0.000, #*p* = 0.000 (anti-VEGF) and *p* = 0.000 (MG + anti-VEGF), &*p* = 0.015; expression levels of **(C)** C3aR mRNA: *p = 0.004, #*p* = 0.014 (MG + anti-VEGF); and **(D)** C3a mRNA: *p = 0.000, #*p* = 0.002 (anti-VEGF) and *p* = 0.000 (MG + anti-VEGF), &*p* = 0.000.

Immunofluorescence findings revealed a rare distribution of C3aR + cells in the healthy retina. After laser injury, they penetrated the whole retina along with the lesion area (Figure 6A). The fluorescence intensity of C3aR + cells significantly reduced after anti-VEGF (*p* = 0.000) or MG + anti-VEGF (*p* = 0.000) treatment compared to the model group. The MG + anti-VEGF group has the lower fluorescence intensity than the anti-VEGF group (*p* = 0.015) (Figure 6B).

According to qRT-PCR analyses, C3aR mRNA expression was significantly lower in the MG + anti-VEGF groups than that in the model group (*p* = 0.014) (Figure 6C). The relative expression level of C3a mRNA showed that both the anti-VEGF (*p* = 0.002) and MG + anti-VEGF groups (*p* = 0.000) were significantly lower than the model group. Compared to the anti-VEGF group, the MG + anti-VEGF group showed a significant decline (*p* = 0.000) (Figure 6D).

### 3.9 Fingerprint analysis of MG

**FIGURE 7:**
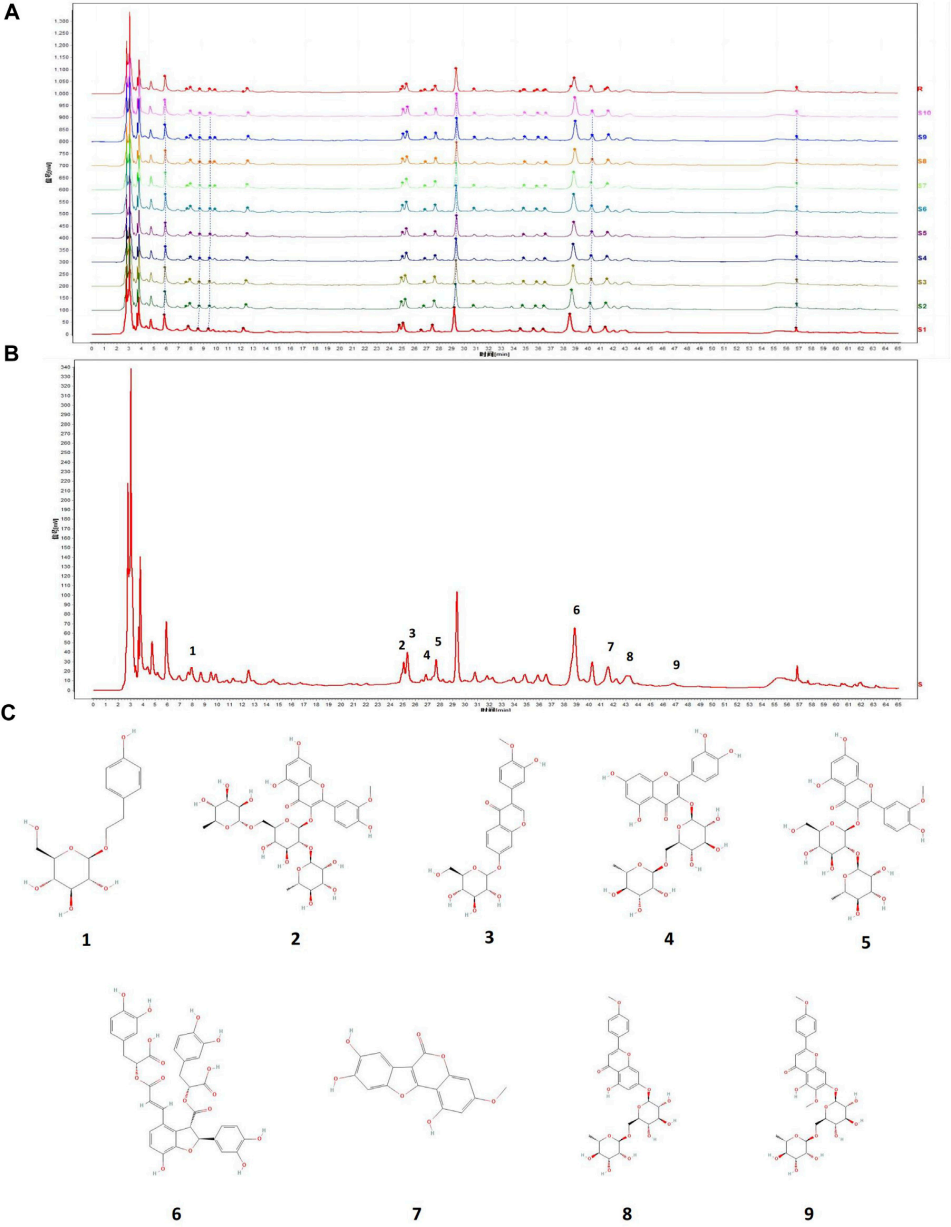
**(A)** Ten batches of fingerprint analysis of MG. **(B)** Nine peaks were confirmed as follows: 1) salidroside, 2) typhaneoside, 3) calycosin-7-glucoside, 4) rutin, 5) isorhamnetin-3-O-neohespeidoside, 6) salvianolic acid B, 7) wedelolactone, 8) linarin, and 9) pectolinarin. **(C)** Chemical structure of metabolites determined in fingerprint.

Ten batches of fingerprint analysis are presented in Figure 7A. A representative fingerprint chromatogram is presented in Figure 7B. A total of 30 common peaks were obtained from the fingerprint spectra of 10 batches of MG and control, and the peak lines and retention times of each batch were relatively consistent (Figure 7A). Among the 10 batches of samples, 8 batches have a similarity of 0.90 or above with the control spectrum, indicating a good product similarity and stable process (Supplementary Material). Nine peaks were identified with comparison to standard metabolites, namely, salidroside, typhaneoside, calycosin-7-glucoside, rutin, isorhamnetin-3-O-neohespeidoside, salvianolic acid B, wedelolactone, linarin, and pectolinarin. The chemical structures of metabolites determined are presented in Figure 7C. Astragaloside IV was not identified in the shared peak and was related to its structure itself.

Typhaneoside and isorhamnetin-3-O-neohespeidoside are characteristic metabolites in *Typha angustifolia* L. (Cao et al., 2015). Wedelolactone is a main metabolite in *Eclipta prostrata* L. (Tian et al., 2023). Linarin is a main metabolite in *Cirsium japonicum* DC. (Ganzera et al., 2005). Rutin is present in *Lycium barbarum* L. (Zhao et al., 2020), and salvianolic acid B is present in *Salvia miltiorrhiza* Bunge (Guo and Wang, 2022).

## 4 Discussion

### 4.1 Summary of evidence

The progression of CNV from a neovascular bundle to a mixed fibrovascular structure, and finally to a scar, is referred to as vascular fibrotic transformation. Fibrotic changes in CNV is a benefit for limitation of exudation, but excessive fibrosis eventually leads to subretinal scarring and makes vision unable to recover (Tenbrock et al., 2022). Anti-VEGF agents are the first-line treatment for nAMD as they can effectively promote the regression of CNV for a short term (Curtis et al., 2012). Unfortunately, the effects of anti-VEGF agents on the recurrence of CNV and macular fibrosis are limited for some patients (Heier, 2009; Daniel et al., 2014). Therefore, we used the two-stage laser to establish a rat model of the subretinal fibrovascular membrane, combined with MG therapy on the basis of an anti-VEGF agent (ranibizumab), to observe the histomorphological therapeutic effect. Our findings showed that the MG + anti-VEGF group had a significant improvement in the CNV exudation area, MD value, fibrovascular membrane lesion height, and local tissue structure damage compared with the simple anti-VEGF treatment. This indicates that this therapy protects the structure of the retina and inhibits the development of CNV and the formation of the fibrovascular membrane.

### 4.2 Mechanism of Mingjing granule on anti-nAMD fibrovascular formation

The formation mechanism of the fibrovascular membrane has not been clarified yet. Our research has preliminarily explored the mechanism of MG from three aspects: inflammation, fibrosis, and complement system. Inflammation is the key factor in the occurrence of nAMD. The inflammatory network composed of inflammatory factors secreted by macrophages, activated microglia, and astrocytes and multiple signaling pathways are involved in the formation of the CNV fibrovascular membrane (Rathnasamy et al., 2019; Gharagozloo et al., 2021). Therefore, reducing inflammatory cells and achieving inhibition of inflammasome activation are the focus of nAMD treatment (Cheung et al., 2019). We used F4/80, Iba-1, and GFAP as markers of macrophages, microglia, and astrocytes, respectively, and detected their distribution and expression levels by immunofluorescence and qRT-PCR to observe whether MG + anti-VEGF inhibits the activation of inflammatory cells. We found that MG + anti-VEGF therapy significantly reduced the expression and distribution of F4/80, Iba-1, and GFAP in the retina and had a certain inhibitory effect on the activation of inflammatory cells. The overall effect was better than that of the anti-VEGF agent alone. Its anti-inflammatory properties may be related to the metabolites in MG. Fan et al. (2018) reported that salvianolic acid B significantly reduced the expression of GFAP and Iba-1 and inhibited the overactivation of astrocytes and microglia in a mouse model of ischemia/reperfusion-induced brain injury. It also reduced the expression of tumor necrosis factor-alpha (TNF-α) and F4/80, which play an anti-inflammatory role in nonalcoholic fatty liver disease (Meng et al., 2022). Similarly, in neurological diseases, rutin alleviated neuroinflammation by reducing the expression of GFAP, interleukin-8, inducible nitric oxide synthase, and nuclear factor κB (Javed et al., 2012; Suganya and Sumathi, 2017).

The expression and regulation level of fibrotic signaling pathways play an important role in the pathogenesis of CNV fibrosis. Fibronectin fragments would stimulate changes in age-related degenerative diseases such as AMD and lead to ocular abnormalities by promoting inflammation, catabolism, and monocyte chemotaxis (Austin et al., 2009). TGF-β1 is a key regulator in the process of fibrosis by activating downstream Smad signaling and the expression of α-SMA, causing tissue fibrosis changes, and collagen-1 is the final effector molecule of soft tissue fibrosis (Cai et al., 2016; Walton et al., 2017). Therefore, reducing scar formation by regulating the expression of the above factors is another direction of current exploration. We found that after the second-stage laser, the positive cells of collagen-1, fibronectin, α-SMA, and TGF-β showed high expression at the lesion. MG + anti-VEGF treatment could reduce their fluorescence intensity (by immunofluorescence). After qRT-PCR analysis of their mRNA expression, it was found that MG + anti-VEGF inhibited the expression of these factors more strongly than the single application of the anti-VEGF agent. We speculate that the mechanism of action is related to MG since multiple metabolites of MG have been shown to inhibit the progression of tissue fibrosis, especially through the TGF-β pathway. Salvianolic acid B has been reported to fight lung and liver fibrosis by inhibiting the overexpression of α-SMA, collagen-1, TGF-β1, and the phosphorylation of Smad3, a downstream target of TGF-β1 (Liu et al., 2016; Jiang et al., 2020). Wedelolactone and rutin also played a similar role. Yang et al. (2019) reported that wedelolactone reversed the increase of bleomycin-induced fibrosis markers collagen-1 and α-SMA, and the decrease of anti-fibrosis markers E-cadherin, and it significantly prevented the phosphorylation of TGF-β1 and Smad2/3. Karunarathne et al. (2023) found that rutin attenuated the gene expression of fibronectin, elastin, collagen-1, and TGF-β, as well as myoblast differentiation from MRC-5 lung fibroblast cells accompanied by the downregulation of α-SMA.

The complement system is equally critical in CNV initiation and fibrosis (Lyzogubov et al., 2014). In the laser-induced nAMD model, the local C3a expression level increased at the CNV lesion, while in the C3-knockout mouse model, the growth of new blood vessels was blocked (Bora et al., 2005). In addition, the combination of complement C3a and C3aR can act on fibroblasts, leading to the occurrence of multi organ fibrosis while enhancing the secretion of effector molecules such as TGF-β (Gu et al., 2016). Lechner et al. (2016) showed that the C3a–C3aR signaling pathway may participate in macular fibrosis of nAMD through the process of inducing fibroblasts, so inhibiting C3a–C3aR may be beneficial to inhibit CNV formation and fibrosis. Our immunofluorescence detection found that C3aR + cells were rarely distributed in the healthy retina, but after laser injury, they penetrated the whole retina with the lesion area. After anti-VEGF treatment, the number of C3aR + cells decreased but still distributed in the whole retina, while after MG + anti-VEGF administration, the fluorescence intensity of C3aR + cells decreased significantly, and the distribution was only found in the ganglion cell layer. qRT-PCR results revealed that MG + anti-VEGF inhibited C3a mRNA expression more strongly than the anti-VEGF agent alone. Previous studies reported that *Salvia miltiorrhiza* significantly reduced the level of complement C3 activation in patients with severe hepatitis, suggesting that *Salvia miltiorrhiza* has a significant inhibitory effect on complement activation (Zhang et al., 1994). Xie et al. (2020) confirmed that *Salvia miltiorrhiza* Bunge has anti-complement activity, with complement C3, C4, and C5 components being its target. *Astragalus mongholicus* Bunge may also reduce the level of complement C3 in diabetic rats (Yang et al., 2010). In conclusion, according to our results, the effect mechanism of MG + anti-VEGF may be related to the fact that the metabolites in MG could inhibit the aggregation of inflammatory cells, control the activation of complement system, and reduce the expression of fibrotic proteins.

### 4.3 Limitations and future directions

The current study verified the protective effect of MG + anti-VEGF therapy on retinal morphology, as well as preliminarily explained its molecular biological mechanism. However, it still has some limitations. This study used ranibizumab, a humanized drug, which resulted in a weaker binding capacity with rat VEGF-A than human VEGF-A; therefore, the results of non-primates may be slightly different from those of humans. We chose a single dose as we had previously explored the effects of different doses of MG on nAMD and determined an optimal dose as the intervention method for this study, which was administered continuously for 40 days. This was a long intervention period, and from an ethical perspective of experimental animals, we followed the 4R rule (reduction, replacement, refinement, and responsibility). If a multi-dose study is established again, it will unnecessarily consume too many animals. In addition, we have also confirmed the efficacy of independent use of MG (Zhou et al., 2014a; Zhou et al., 2014b). One of the purposes of this experiment is to combine MG with an anti-VEGF agent and observe whether they can exert stronger therapeutic effects than a simple anti-VEGF agent, thereby reducing the number of injections for patients; thus, also for animal ethics considerations, we did not establish a separate MG group. Moreover, the pre-clinical pharmacokinetic experiments of MG were not conducted in this study, how it is exposed to the retina enough to induce biological effects are still unclear, so whether the positive biological responses we observed are all/part caused by MG is inconclusive.

Therefore, future studies will compare the therapeutic effects of different doses of MG combined with different injection times of anti-VEGF to determine the best therapeutic regimen and further explain the specific targets and mechanisms of MG in anti-inflammatory, anti-fibrosis, and its effects on the complement system. Meanwhile, pharmacokinetic studies will be carried out to reveal the absorption, distribution, metabolism, and excretion of MG *in vivo*. In addition, the well-designed large sample randomized controlled clinical trials to optimize the application of MG + anti-VEGF therapy in the clinical settings are another direction of future research.

## 5 Conclusion

To sum up, this study indicated that MG + anti-VEGF therapy had an inhibitory effect on the fibrovascular membrane of two-stage laser-induced nAMD rats, possibly mediated by decreasing inflammation, delaying fibrosis process, and regulating activation of the complement system. Our findings suggest that MG could be a promising therapeutic medicament for nAMD. However, well-designed clinical trials are necessary to appraise the efficacy of MG + anti-VEGF therapy in a clinical setting.

## Data Availability

The raw data supporting the conclusion of this article will be made available by the authors, without undue reservation.

## Abbreviations

AMD, age-related macular degeneration; CNV, choroidal neovascularization; FFA, fundus fluorescein angiography; FP, fundus color photograph; GFAP, glial fibrillary acidic protein; H&E, hematoxylin–eosin; Iba-1, ionized calcium-binding adapter molecule 1; MD, mean density; MG, Mingjing granule; nAMD, neovascular AMD; OCT, optical coherence tomography; qRT-PCR, quantitative real-time PCR; RPE, retinal pigment epithelial; TGF-β, transforming growth factor-beta; VEGF, vascular endothelial growth factor; α-SMA, α-smooth muscle actin.
