# Supplementary material for: Mingjing granule inhibits the subretinal fibrovascular membrane of two-stage laser-induced neovascular age-related macular degeneration in rats
Source: Front Pharmacol. 2024 Jun 25;15:1384418. doi: 10.3389/fphar.2024.1384418 (PMC11231192; doi:10.3389/fphar.2024.1384418)
Supplement: Supplementary file 1 [file Table1.DOCX]

| Results of 10 batches of MG fingerprint map similarity evaluation | | | | | | | | | | | |
| --- | --- | --- | --- | --- | --- | --- | --- | --- | --- | --- | --- |
|  | S1 | S2 | S3 | S4 | S5 | S6 | S7 | S8 | S9 | S10 | Control R |
| S1 | 1 | 0.898 | 0.489 | 0.467 | 0.49 | 0.532 | 0.538 | 0.495 | 0.474 | 0.488 | 0.65 |
| S2 | 0.898 | 1 | 0.572 | 0.518 | 0.544 | 0.592 | 0.598 | 0.542 | 0.526 | 0.542 | 0.701 |
| S3 | 0.489 | 0.572 | 1 | 0.964 | 0.957 | 0.977 | 0.977 | 0.965 | 0.975 | 0.976 | 0.968 |
| S4 | 0.467 | 0.518 | 0.964 | 1 | 0.996 | 0.972 | 0.971 | 0.988 | 0.973 | 0.975 | 0.963 |
| S5 | 0.49 | 0.544 | 0.957 | 0.996 | 1 | 0.973 | 0.972 | 0.986 | 0.964 | 0.968 | 0.967 |
| S6 | 0.532 | 0.592 | 0.977 | 0.972 | 0.973 | 1 | 1 | 0.983 | 0.986 | 0.987 | 0.984 |
| S7 | 0.538 | 0.598 | 0.977 | 0.971 | 0.972 | 1 | 1 | 0.983 | 0.984 | 0.985 | 0.985 |
| S8 | 0.495 | 0.542 | 0.965 | 0.988 | 0.986 | 0.983 | 0.983 | 1 | 0.986 | 0.987 | 0.974 |
| S9 | 0.474 | 0.526 | 0.975 | 0.973 | 0.964 | 0.986 | 0.984 | 0.986 | 1 | 0.996 | 0.968 |
| S10 | 0.488 | 0.542 | 0.976 | 0.975 | 0.968 | 0.987 | 0.985 | 0.987 | 0.996 | 1 | 0.973 |
| Control R | 0.65 | 0.701 | 0.968 | 0.963 | 0.967 | 0.984 | 0.985 | 0.974 | 0.968 | 0.973 | 1 |

Among the 10 batches of samples, 8 batches have a similarity of 0.90 or above with the control spectrum, indicating a good product similarity and stable process.
