# Supplementary material for: Mingjing granule inhibits the subretinal fibrovascular membrane of two-stage laser-induced neovascular age-related macular degeneration in rats
Source: Front Pharmacol. 2024 Jun 25;15:1384418. doi: 10.3389/fphar.2024.1384418 (PMC11231192; doi:10.3389/fphar.2024.1384418)
Supplement: Supplementary file 2 [file DataSheet1.docx]

**Supplementary Material 2**

**Fingerprinting methods of Mingjing granules**

**1 Qualitative study**

**1.1 Instruments and reagents**

Chromatographical analysis was conducted on Waters e2695 HPLC platform (Waters Company, USA; PDA Detector, Empower 3 workstation), Methanol was purchased from Beijing Chemical Works, China. Acetonitrile was purchased from Thermo Fisher Scientific Co., Ltd., USA. Phosphoric acid was purchased from Tianjin Guangfu Technology Development Co., Ltd., China. Methanol (analytically pure) was purchased from Beijing chemical industry group Co., Ltd., China.

Mingjing granules (MG) was produced by Beijing Tcmages Pharmaceutical Co., LTD., China, Typha angustifolia L. (20043161), Eclipta prostrata (L.) L. (20016041), Astragalus mongholicus Bunge (20026161), Salvia miltiorrhiza Bunge (20031621), Cirsium japonicum DC. (20025161), Cirsium arvense var. arvense (20016911), Lycium barbarum L. (22016111), Ligustrum lucidum W.T.Aiton (20016941).

Salidroside (PS011472), Typhaneoside (PS000961), Calycosin-7-glucoside (PS000687), Rutin (PS012233), Isorhamnetin-3-O-neohespeidoside (PS011873), Salvianolic acid B (PS012387), Wedelolactone (PS011796), Linarin (PS011370), Pectolinarin (PS000267), Astragaloside IV (PS012327) were purchased from Chengdu Push Bio-technology Co., Ltd., China.

**1.2 Research methods of three orthogonal fingerprint map of Mingjing granules**

**1.2.1 Sample**

30 batches of Mingjing granules samples.

**1.2.2 Chromatographic conditions**

Samples were subjected to separation using Agilent HC-C18 chromatographic column (4.6mm×250mm, 5μm). The mobile phase was acetonitrile (A) and 0.1% phosphoric acid aqueous solution (B), and gradient elution was performed according to the elution procedure in Table 1. The flow rate was 1.0 mL/min, the column temperature was 30 ℃, and the detection wavelength was 254nm.

| Table 1 Gradient elution procedure | | |
| --- | --- | --- |
| Time（min） | Mobile phase A (%) | Mobile phase B (%) |
| 0 | 10 | 90 |
| 15 | 15 | 85 |
| 30 | 25 | 75 |
| 50 | 30 | 70 |
| 55 | 70 | 30 |
| 60 | 100 | 0 |
| 65 | 100 | 0 |

**1.2.3 Preparation of the test article solution**

Sample was accurately weighed and placed in a conical flask with a stopper. The 30 batches of samples were divided into three groups, with 10 batches of samples in each group. 50ml of 100% methanol, 50ml of pure water and 50ml of 70% methanol were accurately added, weighed, ultrasonic treated for 30min, cooled, weighed again, added the weight lost, shaken, filtered, and injected 10μL for determination.

**1.2.4 Fingerprint establishment**

Take 30 batches of Mingjing granule samples, prepare the test solution, analyze according to the chromatographic conditions, import the obtained chromatographic data into the Chinese chromatographic fingerprint similarity evaluation system software (2012 Edition) of the national pharmacopoeia commission, take the S1 sample spectrum as the reference spectrum, use the median method, set the time window to 0.1 min, and obtain the superimposed spectrum and the control fingerprint of each 10 batches of samples after multi-point correction.

**1.2.5 Fingerprint similarity evaluation**

The similarity of each 10 batches of MG samples of the same solvent was evaluated by using the common mode as a control fingerprint.

**1.2.6 Common feature peak identification**

The existing content control indicators in the composition of MG were selected as the indicators, which were Salvianolic acid B, Calycosin-7-glucoside, Linarin, Wedelolactone, Astragaloside IV, Salidroside, Typhaneoside, Pectolinarin, Isorhamnetin-3-O-neohespeidoside, Rutin. Take an appropriate amount of the reference substance, accurately weigh it, and place it in a measuring flask to prepare Wedelolactone, Astragaloside IV, Pectolinarin, Rutin, Salvianolic acid B, Calycosin-7-glucoside, Linarin, Salidroside, Typhaneoside, Isorhamnetin-3-O-neohespeidoside with a concentration of 250μg/ml、250μg/ml、250μg/ml、250μg/ml、200μg/ml、200μg/ml、200μg/ml、200μg/ml、200μg/ml、200μg/ml.

**1.3 Results**

**1.3.1 MG was extracted with 100% methanol solvent**

**1.3.1.1 Fingerprint map and the control map**

Our study identified common peaks of MG and generated a control map (R spectrum). The results showed that 10 batches of MG fingerprint and control fingerprint obtained 30 common peaks, and the peak line and retention time of each batch were relatively consistent, indicating that the similarity of the product was good and the process was stable, see Figure 1.


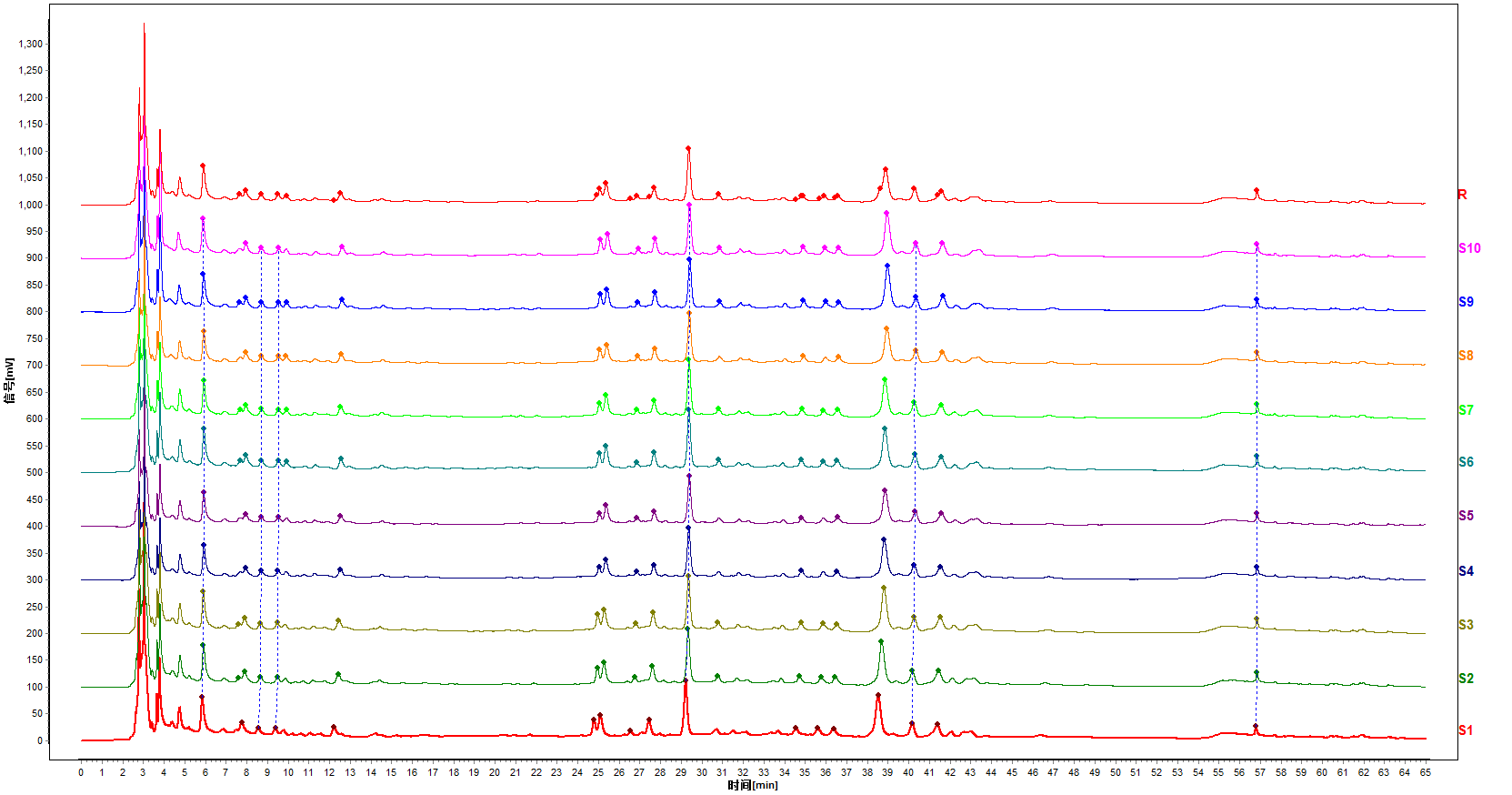


Figure 1 Ten batches of MG fingerprint and control fingerprint were extracted from 100% methanol solvent

**1.3.1.2 Similarity evaluation**

The similarity evaluation results of 10 batches of MG showed that there was some difference in similarity. It is said that there was still some difference between the batches of MG, which can be graded according to the evaluation results of fingerprint similarity.

**1.3.1.3 Common characteristic peak identification**

Nine peaks identified with comparison to standard metabolites, including 1) Salidroside, 2) Typhaneoside, 3) Calycosin-7-glucoside, 4) Rutin, 5) Isorhamnetin-3-O-neohespeidoside, 6) Salvianolic acid B, 7) Wedelolactone, 8) Linarin, 9) Pectolinarin. Astragaloside IV was not identified in the shared peak and was related to its structure itself. See Figure 2 for details.


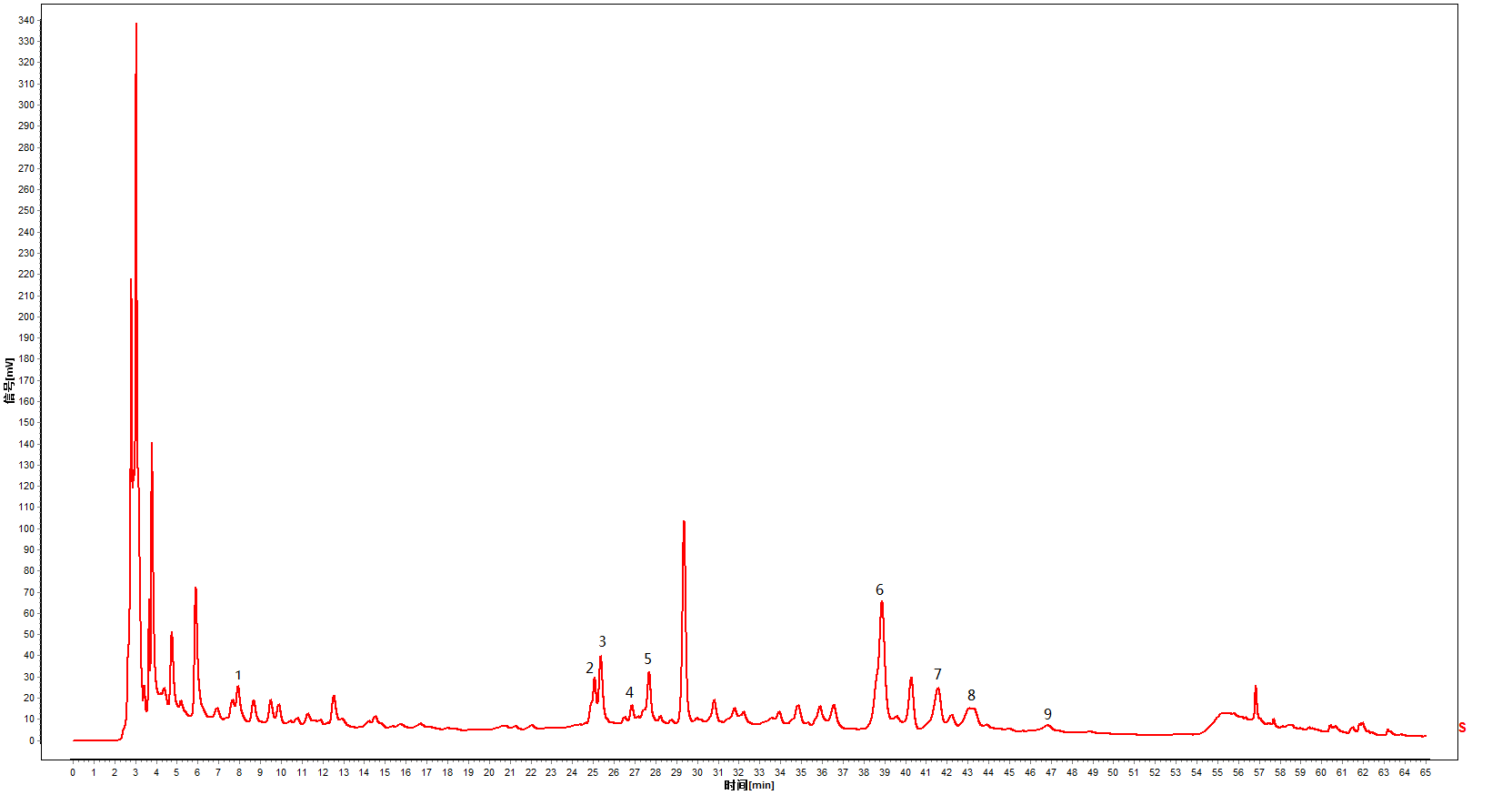


Figure 2 Common characteristic peaks in the HPLC control map. 1) Salidroside, 2) Typhaneoside, 3) Calycosin-7-glucoside, 4) Rutin, 5) Isorhamnetin-3-O-neohespeidoside, 6) Salvianolic acid B, 7) Wedelolactone, 8) Linarin, 9) Pectolinarin.

**1.3.2 MG was extracted with 70% methanol solvent**

**1.3.2.1 Fingerprint map and the control map**

Our study identified common peaks of MG and generated a control map (R spectrum). The results showed that 10 batches of MG fingerprint and control fingerprint obtained 45 common peaks, and the peak line and retention time of each batch were relatively consistent, indicating that the similarity of the product was good and the process was stable, see Figure 3.


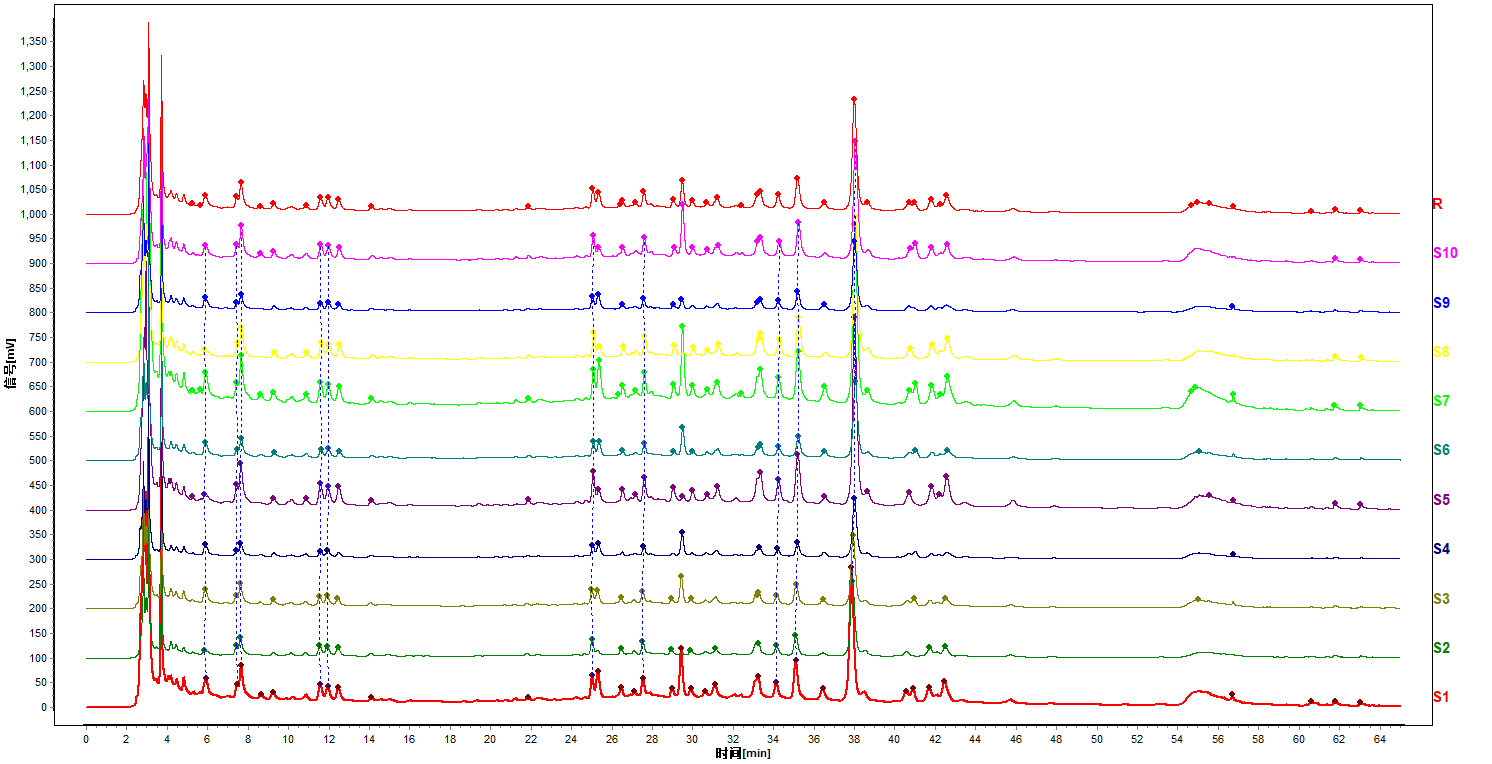


Figure 3 Ten batches of MG fingerprint and control fingerprint were extracted from 70% methanol solvent

**1.3.2.2 Similarity evaluation**

The similarity evaluation results of 10 batches of MG showed that there was some difference in similarity. It is said that there was still some difference between the batches of MG, which can be graded according to the evaluation results of fingerprint similarity.

**1.3.2.3 Common characteristic peak identification**

In the characteristic map, a total of 8 standard substances were recognized, and in the order of time axis, 1) Salidroside, 2) Typhaneoside, 3) Calycosin-7-glucoside, 4) Rutin, 5) Isorhamnetin-3-O-neohespeidoside, 6) Salvianolic acid B, 7) Pectolinarin, 8) Wedelolactone. Linarin and Astragaloside IV were not identified in the shared peak and was related to its structure itself. See Figure 4 for details.


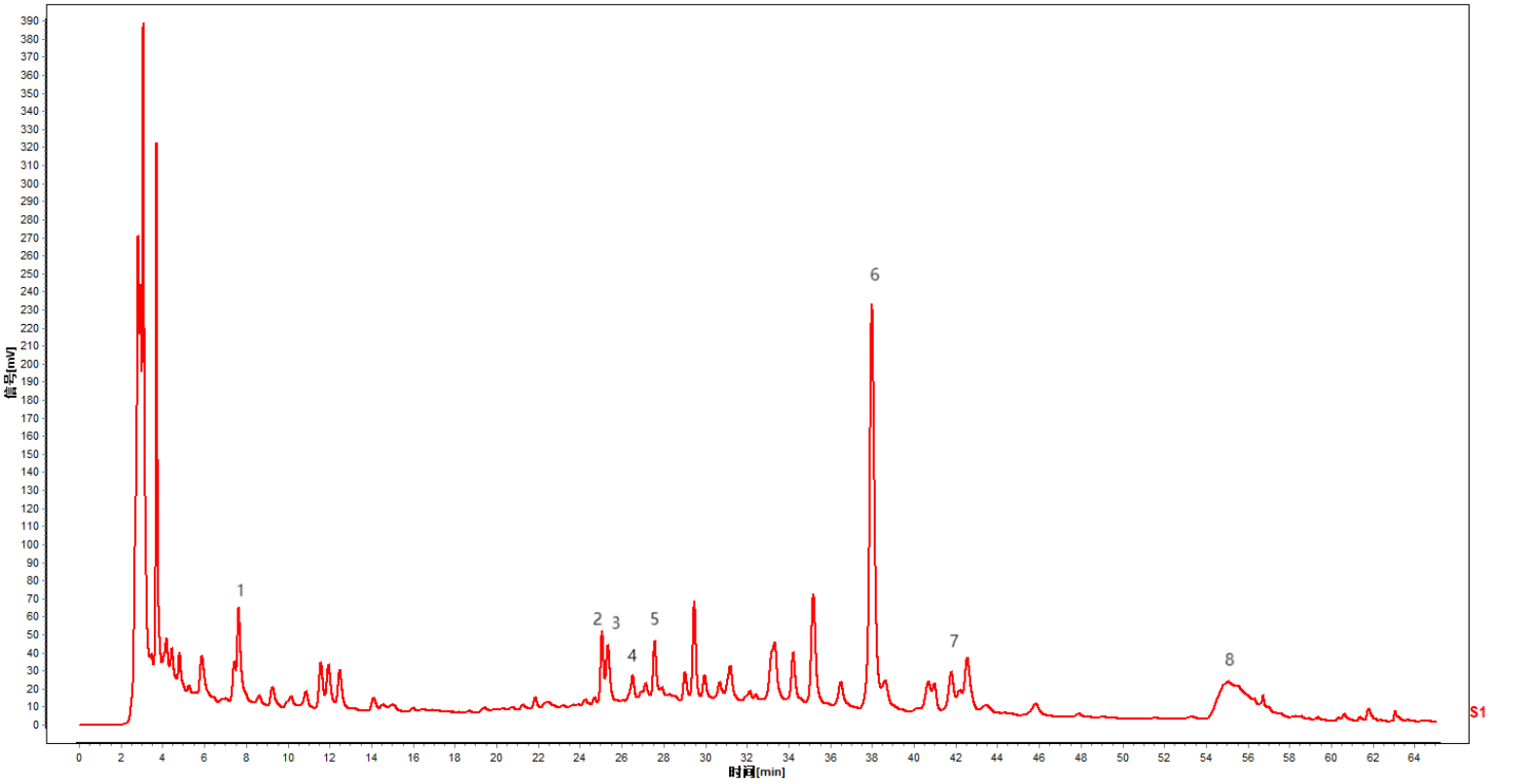


Figure 4 Common characteristic peaks in the HPLC control map. 1) Salidroside, 2) Typhaneoside, 3) Calycosin-7-glucoside, 4) Rutin, 5) Isorhamnetin-3-O-neohespeidoside, 6) Salvianolic acid B, 7) Pectolinarin, 8) Wedelolactone.

**1.3.3 MG was extracted with water**

**1.3.3.1 Fingerprint map and the control map**

Our study identified common peaks of MG and generated a control map (R spectrum). The results showed that 10 batches of MG fingerprint and control fingerprint obtained 21 common peaks, and the peak line and retention time of each batch were relatively consistent, indicating that the similarity of the product was good and the process was stable, see Figure 5.


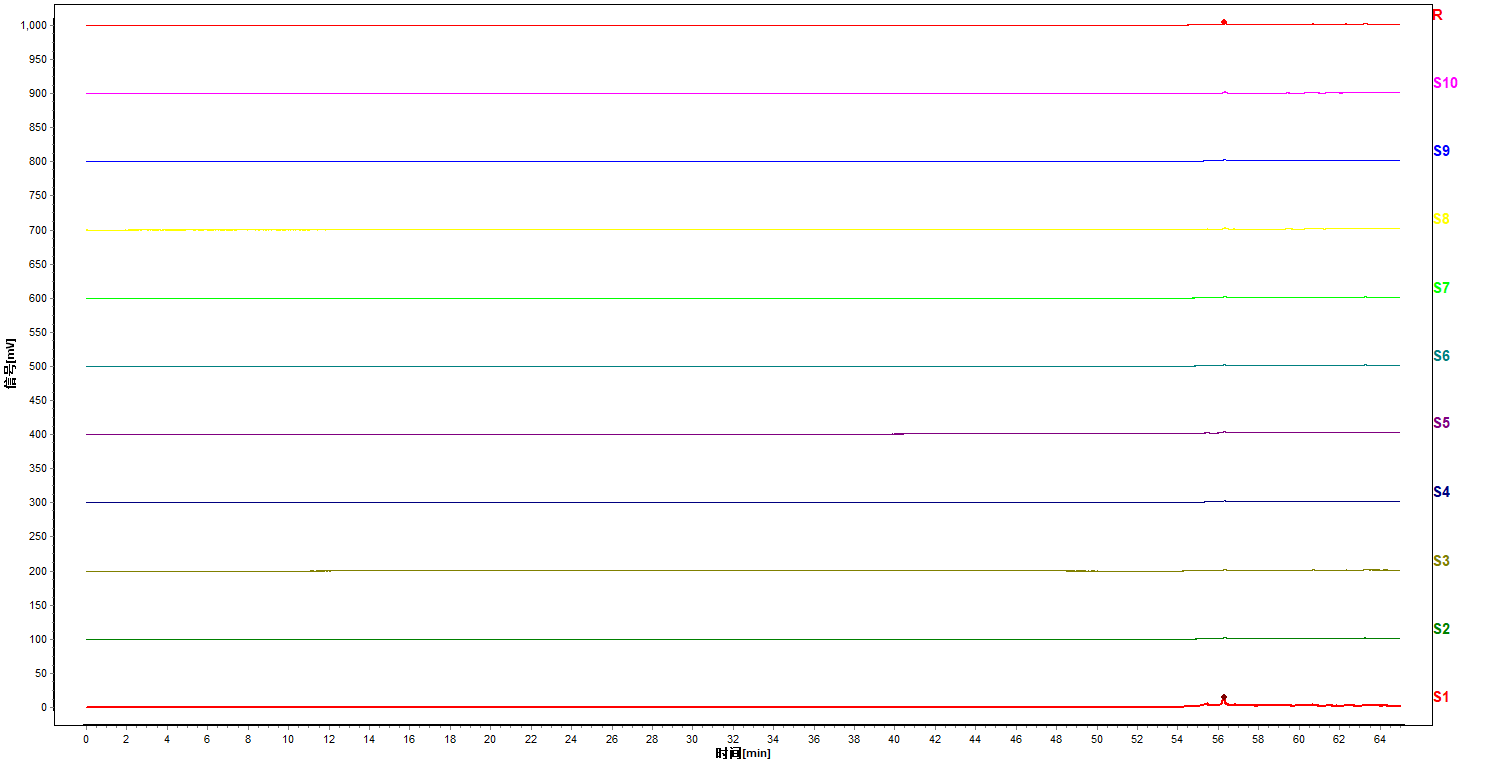


Figure 5 Ten batches of MG fingerprint and control fingerprint were extracted from water

**1.3.3.2 Similarity evaluation**

The similarity evaluation results of 10 batches of MG showed that their similarity was slightly different, which could be classified according to the fingerprint similarity evaluation results.

**1.3.3.3 Common characteristic peak identification**

In the characteristic map, a total of 8 standard substances were recognized, and in the order of time axis, 1) Salidroside, 2) Typhaneoside, 3) Calycosin-7-glucoside, 4) Rutin, 5) Isorhamnetin-3-O-neohespeidoside, 6) Salvianolic acid B, 7) Pectolinarin, 8) Wedelolactone. Linarin and Astragaloside IV were not identified in the shared peak and was related to its structure itself. See Figure 6 for details.


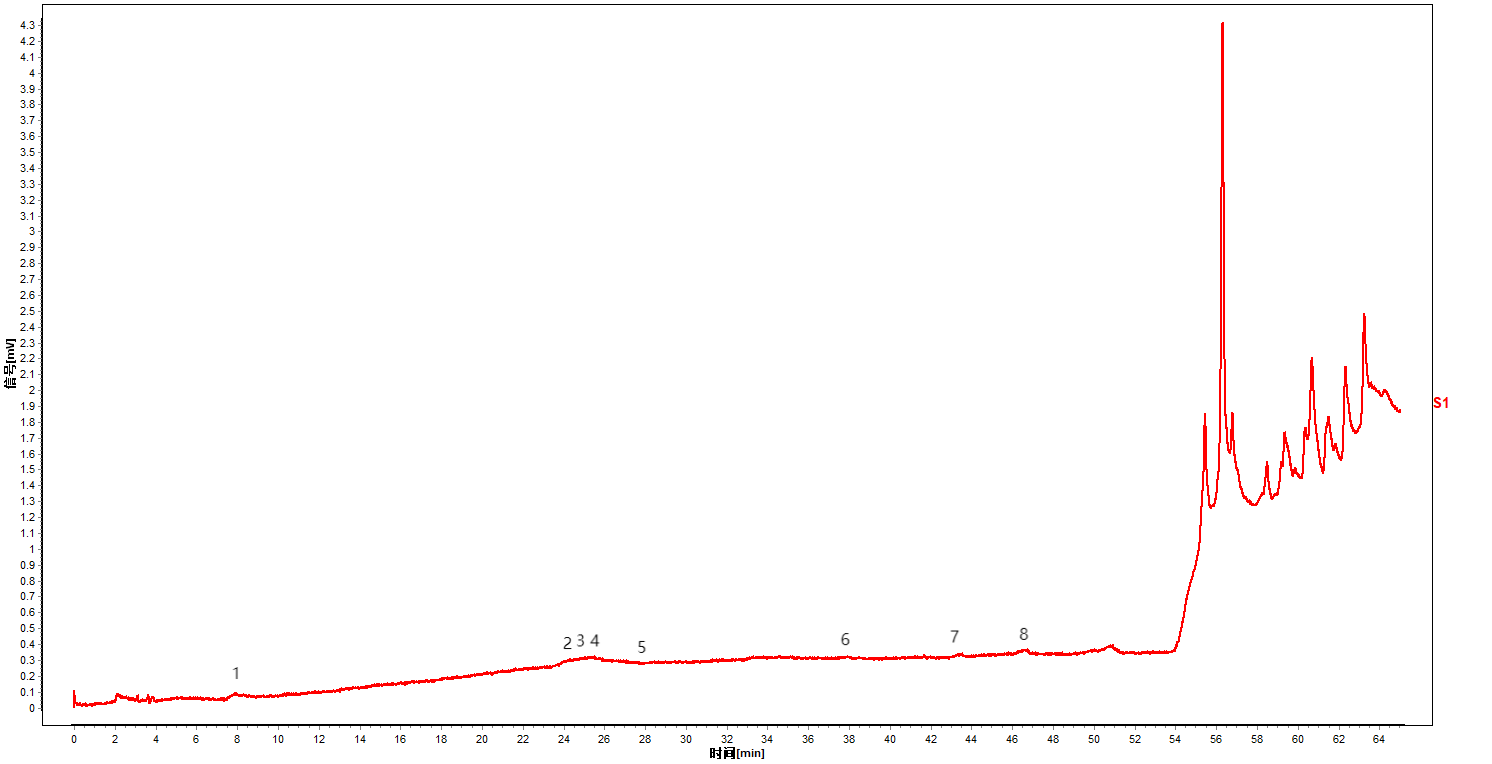


Figure 6 Common characteristic peaks in the HPLC control map. 1) Salidroside, 2) Typhaneoside, 3) Calycosin-7-glucoside, 4) Rutin, 5) Isorhamnetin-3-O-neohespeidoside, 6) Salvianolic acid B, 7) Pectolinarin, 8) Wedelolactone.

**2. Quantitative study**

The content of compounds in Mingjing granules was determined by taking single traditional Chinese medicine formula granules as the unit. There were 8 traditional Chinese medicine formula granules. The content of at least 2 labeled compounds was determined for each traditional Chinese medicine. Take Puhuang formula granules as an example.

**2.1 Preparation method**

Take 3800g of Typha angustifolia L., boil with water, filter, and concentrate the filtrate into a clear paste (with a dry extract yield of 13.2%~19.3%). Add an appropriate amount of excipients, dry (or crush), then add an appropriate amount of excipients, mix well, and granulate to make 1000g.

**2.2 Specific chromatogram**

Determine according to the high-performance liquid chromatography method (General Rule 0512 of the 2020 edition of the Chinese Pharmacopoeia).

**2.2.1 Preparation of reference solution**

Take 0.5g of Typha angustifolia L. reference material, put it into a corked conical flask, add 25ml of water, heat and reflux for 60 minutes, cool it, shake it well, filter it, and take the filtrate as the reference solution of the reference material. In addition, take an appropriate amount of Isorhamnetin-3-O-neohespeidoside reference, Typhaneoside, Quercetin 3-O-neohesperidoside reference, Kaempferol 3-O-neohesperidoside reference, accurately weigh, and add methanol to prepare a solution containing 50µg Isorhamnetin-3-O-neohespeidoside, 50µg Typhaneoside, 40µg Quercetin 3-O-neohesperidoside, and 20µg Kaempferol 3-O-neohesperidoside per 1 ml as the reference solution of the control.

**2.2.2 Determination method**

Precisely suck 1µl of the test solution and 1µl of the reference solution, inject them into the ultra performance liquid chromatograph, and determine them.

Eight characteristic peaks should be present in the chromatography of the test article and should correspond to the retention time of the eight characteristic peaks in the reference sample. Among them, peaks 4 and 6 to 8 correspond to the retention time of Quercetin 3-O-neohesperidoside, Typhaneoside, Kaempferol 3-O-neohesperidoside, and Isorhamnetin-3-O-neohespeidoside reference, respectively, and the peak corresponding to the peak of Typhaneoside reference is S peak. Calculate the relative retention time of peak 3, peak 5 and peak s, and the relative retention time shall be within ± 10% of the specified value. The specified values are: 0.86 (peak 3), 0.98 (peak 5). See Figure 7 for details.


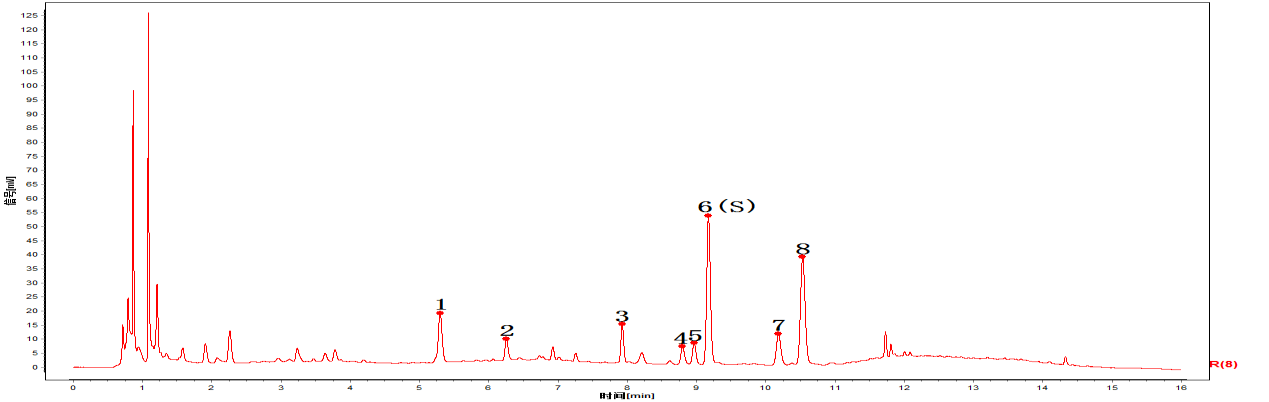


Figure 7 Comparison characteristic map. Peak 1: 4-Hydroxybenzoic acid, peak 2: Vanillic acid, peak 4: Quercetin 3-O-neohesperidoside, peak 6 (S): Typhaneoside, peak 7: Kaempferol 3-O-neohesperidoside, peak 8: Isorhamnetin-3-O-neohespeidoside. Chromatographic column: ZORBAX Eclipse Plus C18，2.1mm×100mm，1.8μm

**2.2.3 Inspect**

Comply with relevant provisions under granules (general rule 0104 of Chinese Pharmacopoeia 2020 Edition).

**2.2.4 Extract**

According to the determination method of alcohol soluble extract (general rule 2201 of Chinese Pharmacopoeia 2020 Edition), using ethanol as solvent, it shall not be less than 15.0%.

**2.2.5 HPLC**

**2.2.5.1 Chromatographic conditions and system suitability test**

Octadecylsilane bonded silica gel was used as filler (column length: 100mm, inner diameter: 2.1mm, particle size: 1.8μm) ; Using acetonitrile as mobile phase A and 0.1% acetic acid solution as mobile phase B, perform gradient elution as specified in the following Table 2; The flow rate was 0.3ml per minute; The column temperature was 30 ℃; The detection wavelength was 254nm. The number of theoretical plates shall not be less than 5000 based on isorhamnetin-3-o-neohesperidin peak.

| Table 2 Gradient elution procedure | | |
| --- | --- | --- |
| Time（min） | Mobile phase A (%) | Mobile phase B (%) |
| 0~3 | 5→10 | 95→90 |
| 3~5 | 10→16 | 90→84 |
| 5~9 | 16→18 | 84→82 |
| 9~14 | 18→51 | 82→49 |
| 14~16 | 51→5 | 49→95 |

**2.2.5.2 Preparation of reference solution**

Take an appropriate amount of Isorhamnetin-3-O-neohespeidoside reference substance and Typhaneoside reference substance, accurately weigh them, and add methanol to make a mixed solution containing 40µg per 1ml.

**2.2.5.3 Preparation of test solution**

Take an appropriate amount of this product, grind it into fine powder, take about 0.2g, weigh it accurately, place it in a conical flask with a stopper, add 25ml of 30% methanol precisely, close the stopper, weigh it, conduct ultrasonic treatment (power 250W, frequency 40KHz) for 30 minutes, cool it, weigh it again, make up the lost weight with 30% methanol, shake it well, filter it, and take the filtrate.

**2.2.5.4 Determination method and results**

Precisely suck 1µl of the reference solution and 1µl of the test solution respectively, inject them into the ultra performance liquid chromatograph, and determine them.

The total amount of Isorhamnetin-3-O-neohespeidoside (C28H32O16) and Typhaneoside (C34H42O20) per 1g of this product should be 2.5mg~11.0mg.

**2.2.6 Specifications**

Each 1g of Puhuang formula granule is equivalent to 3.8g of decoction pieces.
